# Phylogenomic Diversity Elucidates Mechanistic Insights into Lyme Borreliae-Host Association

**DOI:** 10.1128/msystems.00488-22

**Published:** 2022-08-08

**Authors:** Matthew Combs, Ashley L. Marcinkiewicz, Alan P. Dupuis, April D. Davis, Patricia Lederman, Tristan A. Nowak, Jessica L. Stout, Klemen Strle, Volker Fingerle, Gabriele Margos, Alexander T. Ciota, Maria A. Diuk-Wasser, Sergios-Orestis Kolokotronis, Yi-Pin Lin

**Affiliations:** a Department of Ecology, Evolution, and Environmental Biology, Columbia University, New York, New York, USA; b Division of Infectious Diseases, Wadsworth Center, New York State Department of Health, Albany, New York, USA; c Department of Biomedical Sciences, SUNY Albany, Albany, New York, USA; d German National Reference Centre for Borrelia, Bavarian Health and Food Safety Authority, Oberschleissheim, Germany; e Department of Epidemiology and Biostatistics, School of Public Health, SUNY Downstate Health Sciences University, Brooklyn, New York, USA; f Institute for Genomics in Health, SUNY Downstate Health Sciences University, Brooklyn, New York, USA; g Division of Infectious Diseases, Department of Medicine, College of Medicine, SUNY Downstate Health Sciences University, Brooklyn, New York, USA; h Department of Cell Biology, College of Medicine, SUNY Downstate Health Sciences University, Brooklyn, New York, USA; University of California, San Diego

**Keywords:** host association, *Borrelia*, complement, phylogenomics, plasmid diversity

## Abstract

Host association—the selective adaptation of pathogens to specific host species—evolves through constant interactions between host and pathogens, leaving a lot yet to be discovered on immunological mechanisms and genomic determinants. The causative agents of Lyme disease (LD) are spirochete bacteria composed of multiple species of the Borrelia burgdorferi *sensu lato* complex, including B. burgdorferi (*Bb*), the main LD pathogen in North America—a useful model for the study of mechanisms underlying host-pathogen association. Host adaptation requires pathogens’ ability to evade host immune responses, such as complement, the first-line innate immune defense mechanism. We tested the hypothesis that different host-adapted phenotypes among *Bb* strains are linked to polymorphic loci that confer complement evasion traits in a host-specific manner. We first examined the survivability of 20 *Bb* strains in sera *in vitro* and/or bloodstream and tissues *in vivo* from rodent and avian LD models. Three groups of complement-dependent host-association phenotypes emerged. We analyzed complement-evasion genes, identified *a priori* among all strains and sequenced and compared genomes for individual strains representing each phenotype. The evolutionary history of *ospC* loci is correlated with host-specific complement-evasion phenotypes, while comparative genomics suggests that several gene families and loci are potentially involved in host association. This multidisciplinary work provides novel insights into the functional evolution of host-adapted phenotypes, building a foundation for further investigation of the immunological and genomic determinants of host association.

**IMPORTANCE** Host association is the phenotype that is commonly found in many pathogens that preferential survive in particular hosts. The Lyme disease (LD)-causing agent, B. burgdorferi (*Bb*), is an ideal model to study host association, as *Bb* is mainly maintained in nature through rodent and avian hosts. A widespread yet untested concept posits that host association in *Bb* strains is linked to *Bb* functional genetic variation conferring evasion to complement, an innate defense mechanism in vertebrate sera. Here, we tested this concept by grouping 20 *Bb* strains into three complement-dependent host-association phenotypes based on their survivability in sera and/or bloodstream and distal tissues in rodent and avian LD models. Phylogenomic analysis of these strains further correlated several gene families and loci, including *ospC*, with host-specific complement-evasion phenotypes. Such multifaceted studies thus pave the road to further identify the determinants of host association, providing mechanistic insights into host-pathogen interaction.

## INTRODUCTION

Infectious disease systems are governed by the evolution of host-pathogen interactions. Some pathogens can survive in multiple host species, but tend to preferentially adapt to some hosts over others—a process known as host association (1). Although this is an attractive theory, the mechanisms and genetic basis of host association remain largely unexplored. The genospecies complex of the bacterial spirochete Borrelia burgdorferi *sensu lato* (also known as Borreliella burgdorferi *sensu lato* or Lyme borreliae) are the causative agents of Lyme disease, the most common vector-borne disease in North America and Europe (2, 3). Transmitted by a generalist Ixodes tick and carried by multiple vertebrate host species, the Lyme disease bacterium is a suitable model to study the mechanisms that mediate host association (1, 4). In fact, field and laboratory evidence suggest that different Lyme borreliae species vary in their host association (1, 2, 4, 5). For example, birds and rodents are the most common reservoir hosts that are selectively associated with Borrelia garinii and Borrelia afzelii, respectively, two frequently observed Lyme borreliae species in Eurasia (1, 4). In contrast, both host types were found to carry B. burgdorferi *sensu stricto* (here B. burgdorferi), the most commonly isolated Lyme borreliae species in North America (1, 4). However, B. burgdorferi exhibits extensive strain diversity with different genotypes defined by several polymorphic loci (e.g., ribosomal RNA intergenic spacer type [RST], multilocus sequence type [MLST], *ospC*) (6–9), and rodent or bird host associations have been documented in some genotypes of this spirochete species (5, 10, 11). Thus, although still under debate, B. burgdorferi strains with different genotypes appear to exhibit variable host association.

To invade a host, Lyme borreliae require the ability to initially colonize and replicate at the tick bite site, migrate from those sites to the bloodstream, and subsequently disseminate to distal tissues (2, 12). In humans, systemic infections cause multiple manifestations in heart, joints, and neurological tissues, but in reservoir animals, the spirochetes persist at those distal tissues and organs without triggering symptoms (12, 13). Systemic spread requires the mechanisms that facilitate hematogenous dissemination. As the first line of the host immune mechanism present in the blood, complement has been shown to control the ability of Lyme borreliae to disseminate to distal tissues (14–16), emphasizing the role of this immune response in potentially dictating host association of Lyme borreliae.

Complement is a cascade comprised of several serum proteins, which can be activated by three canonical pathways (i.e., classical, alternative, and lectin pathways), resulting in digestion of these proteins to form different protein complexes (17). The activation of complement leads to the release of complement peptides, resulting in inflammation and phagocytosis. That activation also causes the deposition of several complement proteins (C5b, C6, C7, C8, and C9) that generate a pore-forming membrane attack complex (C5b-9) on the pathogen surface to lyse pathogens (17). In the absence of pathogens, vertebrate hosts produce complement regulatory proteins, such as factor H (FH), inhibiting the complement to prevent host cell damages (18, 19). Similar to other pathogens, Lyme borreliae equip their surface with a group of anti-complement proteins to survive in the serum of the blood, known as serum resistance/serum survivability (15, 16, 20). These proteins include CspA (a member of the protein family 54, Pfam54), CspZ, and OspE paralogs (collectively known as complement regulator acquiring surface proteins [CRASPs]) that bind to FH to inactivate the alternative complement pathway (21). Additionally, the spirochete proteins *bb_k32* and Elp paralogs bind to C1, whereas the other spirochete protein, OspC, binds to C4b. These proteins block the activation of the classical complement pathway, whereas OspC also inactivates the lectin complement pathway by binding to respective complement components (22–25). Therefore, this variety of spirochete surface-localized, anti-complement proteins suggests that these proteins, if polymorphic, confer Lyme borreliae species- or strain-specific host association. In fact, CspA is highly polymorphic among the variants from different Lyme borreliae species, and the allelic variation of this protein promotes rodent- or bird-specific complement evasion and spirochete transmission via tick feeding (26).

While divergent across Lyme borreliae species, the CspA variants from different strains within the same species (e.g., B. burgdorferi, B. afzelii) are highly conserved (~99% sequence identity) (26). However, comparative analysis of B. burgdorferi genomes revealed a variable plasmid content and extensive protein-coding polymorphism (26–28). These findings suggest that polymorphisms and the presence or absence of Lyme borreliae proteins with critical contributions to infectivity (e.g., anti-complement proteins) may confer B. burgdorferi strain-specific host association. To address this, we investigated the following: (i) Do different B. burgdorferi strains exhibit variable host-specific complement evasion phenotypes, and do they correlate with phylogenetic divergence of anti-complement proteins? (ii) If so, do host-specific complement evasion phenotypes among strains link to distinct host-specific infectivity (i.e., tissue dissemination)? (iii) Does genomic variation among strains with host-specific complement evasion phenotypes provide additional insights into mechanisms of host association? In this study, we examined the ability of multiple genotypically distinct B. burgdorferi strains to evade the complement from laboratory rodent and avian model animals, Mus musculus mouse and coturnix quail, respectively, and promote infectivity in these hosts. We contextualized the results with phylogenetic analysis of anti-complement genes and comparative genomic analyses from these B. burgdorferi strains to provide mechanistic insights into Lyme borreliae-host association.

## RESULTS

### Phylogenetic association of B. burgdorferi host-dependent anti-complement phenotypes.

We first examined whether genotypically distinct B. burgdorferi strains display different levels of complement evasion phenotypes in a host-dependent manner and whether such differences are associated with the phylogeny of the anti-complement proteins from each of these strains. We thus chose 20 B. burgdorferi strains with different genotypes based on typing different polymorphic loci (i.e., *ospC*, RST, and MLST) (Table 1).

**TABLE 1:** The genotypes and anti-complement phenotypes of B. burgdorferi strains used in this study^*a*^

Strains	Genotype			Geographical location	Source	Anti-complement phenotypes^*b*^		Accession no. of genome	References of source
	MLST	RST	OspC						
						Rodent^*c*^	Quail		
B31A^*d*^	1	1	A	North America (New York, USA)	Tick (I. scapularis)	−	−	n.a.	(92)
B313^*e*^	1	1	A	North America (New York, USA)	Tick (I. scapularis)	−	−	n.a.	(93)
B31-ref^*f*^	1	1	A	North America (New York, USA)	Tick (I. scapularis)	+	+	GCA_000008685.2	(74, 94)
B31-5A4	1	1	A	North America (New York, USA)	Tick (I. scapularis)	+	+	CP094597-CP094617	(74, 94)
PAbe	1	1	A	Europe (Germany)	Human (CSF)	+	+	GCA_002151485.1	(93)
PAli	1	1	A	Europe (Germany)	Human (skin)	+	+	GCA_002151465.1	(95)
BL206	1	1	A	North America (New York, USA)	Human (blood)	+	+	JALGSN010000000	(56)
ZS7	20	1	B	Europe (Germany)	Tick (*I. ricinus*)	+	+	GCA_000021405.1	(96)
PMeh	20	1	B	Europe (Germany)	Human (joints)	+	+	SRX828288	(96)
297	3	2	K	North America (Connecticut, USA)	Human (CSF)	+	−	JALGSQ010000000	(97, 98)
B379	3	2	K	North America (New York, USA)	Human (skin)	−	+	CP094579-CP094596	(99)
Bbss62 (Bbss62.h.ks)	4	2	H	North America (Massachusetts, USA)	Human (skin)	+	+	SAMN29767977	This study
WI91-23	228	3	I	North America (Wisconsin, USA)	Song Sparrow	−	+	GCA_000181855.2	(100)
B408	16	3	I	North America (New York, USA)	Human (skin)	+	−	CP094561-CP094578	(99)
B331	16	3	I	North America (New York, USA)	Human (skin)	+	−	GCA_002442595.1	(99)
29805	12	3	M	North America (Connecticut, USA)	Tick (I. scapularis)	−	+	GCA_000172295.2	(100)
B356	12	3	M	North America (New York, USA)	Human (skin)	−	+	JALGSP010000000	(101, 102)
N40-D10/E9	12	3	M	North America (New York, USA)	Tick (I. scapularis)	+	+	JALGSO010000000	(103)
CA11.2A	333	3	D	North America (California, USA)	Tick (I. pacificus)	+	+	GCA_000172315.2	(104)
MM1	328	3	U	North America (Minnesota, USA)	WF mouse	+	−	GCA_003367295.1	(105)
JD1	11	3	C	North America (Massachusetts, USA)	Tick (I. scapularis)	+	−	GCA_000166655.2	(106)
cN40	19	3	E	North America (New York, USA)	Tick (I. scapularis)	+	+	GCA_000166635.2	(107)

a CSF, Cerebrospinal fluid. MLST, Multilocus sequence type. RST, ribosomal RNA intergenic space type. Not available because these strains were not included in the phylogenetic analysis. WF, white-footed.

b The results were derived from Fig. 1. +, defined by the strains with greater than the threshold values (recruited significantly greater levels of mouse C5b-9 or quail C8 on the surface in the presence of indicated sera than B31-5A4 and had survival percentage greater than 50% in white-footed mouse or quail sera). Otherwise, those strains are shown as “- ” to indicate anti-complement phenotypes.

c Mouse sera for complement deposition assays and white-footed mouse sera for serum resistance assays.

d High-passage B. burgdorferi B31 missing lp21, lp25, lp28-1, lp28-3, lp28-4, lp36, cp9, cp32-6, cp32-8.

e High-passage B. burgdorferi B31 missing lp5, lp17, lp21, lp25, lp28-1, lp28-2, lp28-3, lp28-4, lp36, lp38, lp54, lp56, cp9, cp32-4, cp32-6, cp32-8, cp32-9.

f Reference strain of B. burgdorferi obtained from ATCC (ATCC 35210).

**(i) B. burgdorferi displays strain-level and host-specific variation in complement inactivation and serum resistance.** We first added each of these strains, along with a serum-sensitive and complement-susceptible B. burgdorferi strain B313 (control) into the tested animal sera. These sera included the sera from BALB/c house mice (Mus musculus) and common quail (Coturnix coturnix), the rodent and avian models in the Lyme disease system, respectively (26). We then evaluated the surface deposition levels of mouse C5b-9 and quail C8 that are involved in bacterial lysis as the readout of complement activation. The deposition of mouse C5b-9 and quail C8 was apparent in B313, as expected (172 and 105 MFI for C5b-9 and quail C8 deposition, respectively) (Fig. 1A to D), in agreement with the inability of this strain to inactivate mouse and quail complement (29, 30). In contrast, the levels of the deposition for these complement proteins were close to undetectable on the surface of strain B31-5A4 (6 and 5 MFI for C5b-9 and quail C8 deposition, respectively), consistent with the fact that B31-5A4 inactivates both mouse and quail complement efficiently (29). We also found that the rest of the tested strains can be grouped according to their extent of complement deposition compared with that from B31-5A4 (Fig. 1A to D): (i) The strains displayed indistinguishable levels of deposition for both mouse C5b-9 and quail C8, suggesting their versatile ability to evade complement from both hosts (B31ref, PAbe, PAli, BL206, ZS7, PMeh, Bbss62, N40-D10/E9, CA11.2A, and cN40), similar to B31-5A4 (Fig. 1C and D, red bars); (ii) the strains exhibited significantly higher levels of mouse C5b-9 but not quail C8, suggesting the ability of the quail-specific complement evasion by these strains (B379, WI91-23, 29805, and B356) (Fig. 1C and D, green bars); and (iii) the strains displayed significantly higher levels of quail C8 but not mouse C5b-9, suggesting mouse-specific complement evasion by these strains (297, B408, B331, MM1, and JD1) (Fig. 1C and D, blue bars).

**FIG 1:**
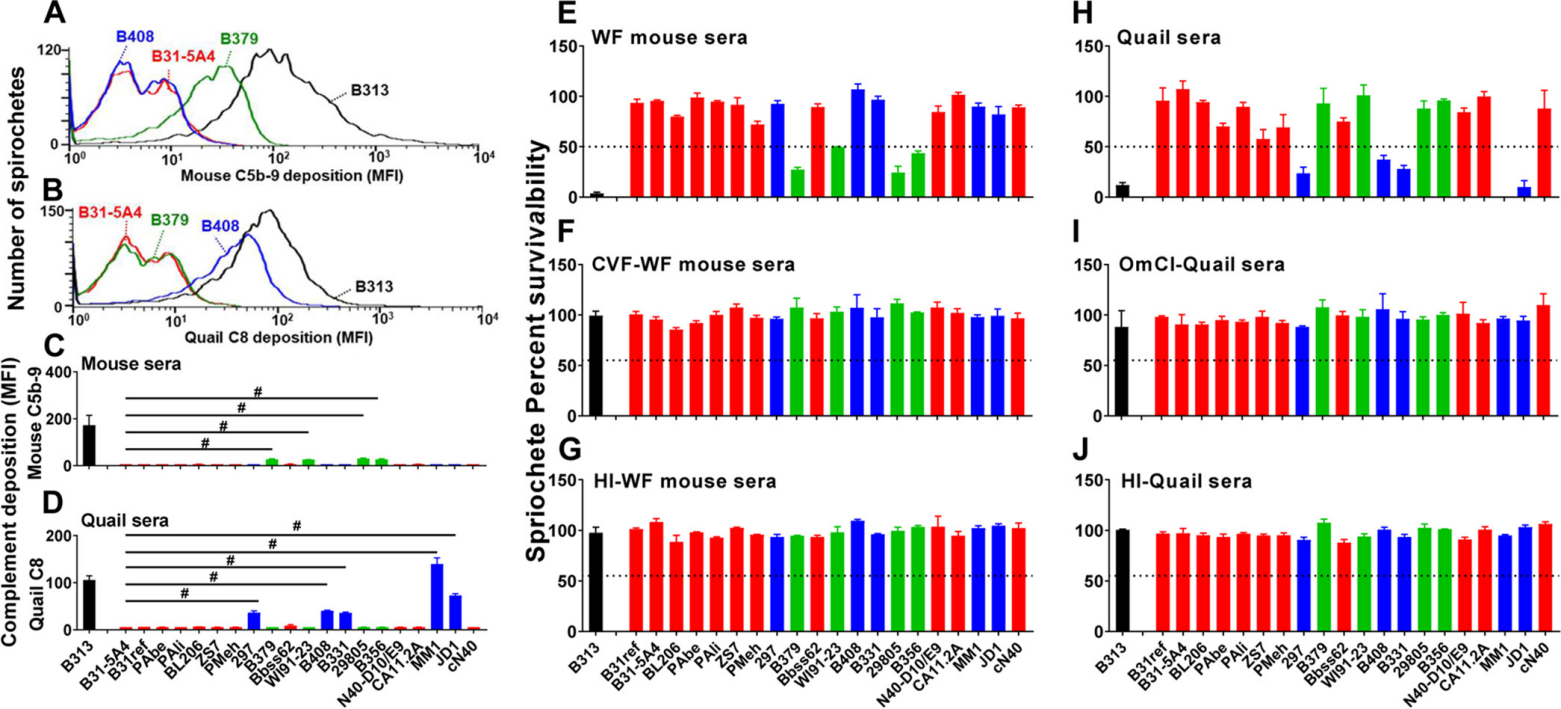
B. burgdorferi displays strain-to-strain variable ability of mammalian and avian serum resistance and complement inactivation. (A to D) Each of the indicated B. burgdorferi strains or the high-passage, noninfectious, and serum-sensitive B. burgdorferi strain B313 (control) was incubated with serum from mouse or quail with a final concentration of 20%. The bacteria were stained with the antibodies that recognize mouse C5b-9 or quail C8 prior to being applied to flow cytometry analysis as described in the Materials and Methods section. Shown are representative histograms of flow cytometry analysis presenting the deposition levels of mouse C5b-9 (A) or quail C8 (B) on the surface of the indicated B. burgdorferi strains. The deposition levels of mouse C5b-9 (C) or quail C8 (D) on the surface of B. burgdorferi were measured by flow cytometry and presented as mean fluorescence index (MFI). Each bar represents the mean of three independent determinations ± standard error of the mean [SEM]. Significant differences (*P* < 0.05, Kruskal-Wallis test with the two-stage step-up method of Benjamini, Krieger, and Yekutieli) in the deposition levels of mouse C5b-9 or quail C8 relative to the B31-5A4 are indicated (#). (E to J) Indicated B. burgdorferi strains were incubated for 4 h with untreated sera from white-footed (WF) mice (E) or quail (H), cobra venom factor (CVF)-treated white-footed mouse sera (F), O. moubata complement inhibitor (OmCI)-treated qual sera (I), or heat-inactivated (HI) sera from white-footed mice (G) or quail (J). The number of motile spirochetes was assessed microscopically. The percentage of survival for those B. burgdorferi strains was calculated using the number of mobile spirochetes at 4 h postincubation normalized to that prior to the incubation with serum. Each bar represents the mean of three independent determinations ± SEM. The black dotted lines indicate the threshold of percentage of survivability (50%). The bars are color coded to represent strains that can efficiently inactivate complement from mouse (blue), quail (green), or both hosts (red) (C to D) or result in more than 50% survivability in the sera from white-footed mice (blue), quail (green), or both hosts (red) (E to J).

We then examined the ability of these strains to survive in complement-containing sera of the white-footed mouse (Peromyscus leucopus), the natural reservoir host of B. burgdorferi in North America, and quail sera. Note that the sera from white-footed rather than BALB/c mice were used to represent rodent sera because the complement in M. musculus sera (i.e., BALB/c mouse sera) is labile *in vitro* (31, 32). As expected, 3 and 12% of B313 survived in white-footed mice and quail sera, respectively, consistent with this strain as serum-sensitive to both sera reported in recent studies (Fig. 1E to J) (11, 30). The survivability of all tested strains varies, ranging from 1.7 to 99.01%. To compare the strain’s ability in surviving in white-footed mouse or quail sera, we set up an arbitrary threshold of survivability at the middle (50%) (black dotted lines in Fig. 1E to J). Such a threshold allows us to group those strains into (i) the strains that survived in both white-footed mouse and quail sera at levels ≥50% (B31ref, B31-5A4, PAbe, PAli, BL206, ZS7, PMeh, Bbss62, N40-D10/E9, CA11.2A, and cN40) (Fig. 1E and H, red bars); (ii) the strains that survived in quail but not in white-footed mouse sera at levels ≥50% (B379, WI91-23, 29805, and B356) (Fig. 1E and H, green bars); and (iii) the strains that survived in white-footed mouse but not in quail sera at levels ≥50% (297, B408, B331, MM1, and JD1) (Fig. 1E and H, blue bars). All of the tested strains survived in complement-inactivated sera (white-footed mouse sera treated with cobra venom factor (CVF) and quail sera treated with Ornithodorus moubata complement inhibitor [OmCI]) at levels greater than 90% (Fig. 1F, G, I, and J). These results of grouping by serum survivability match the grouping by the levels of complement deposition showing three different complement evasion phenotypes for any tested strains (Table 1). Overall, these findings indicate a strain-specific ability to survive in white-footed mouse or quail sera and to prevent complement deposition, grouping genotypically distinct Lyme borreliae strains based on their host-specific anti-complement phenotypes.

**FIG 2:**
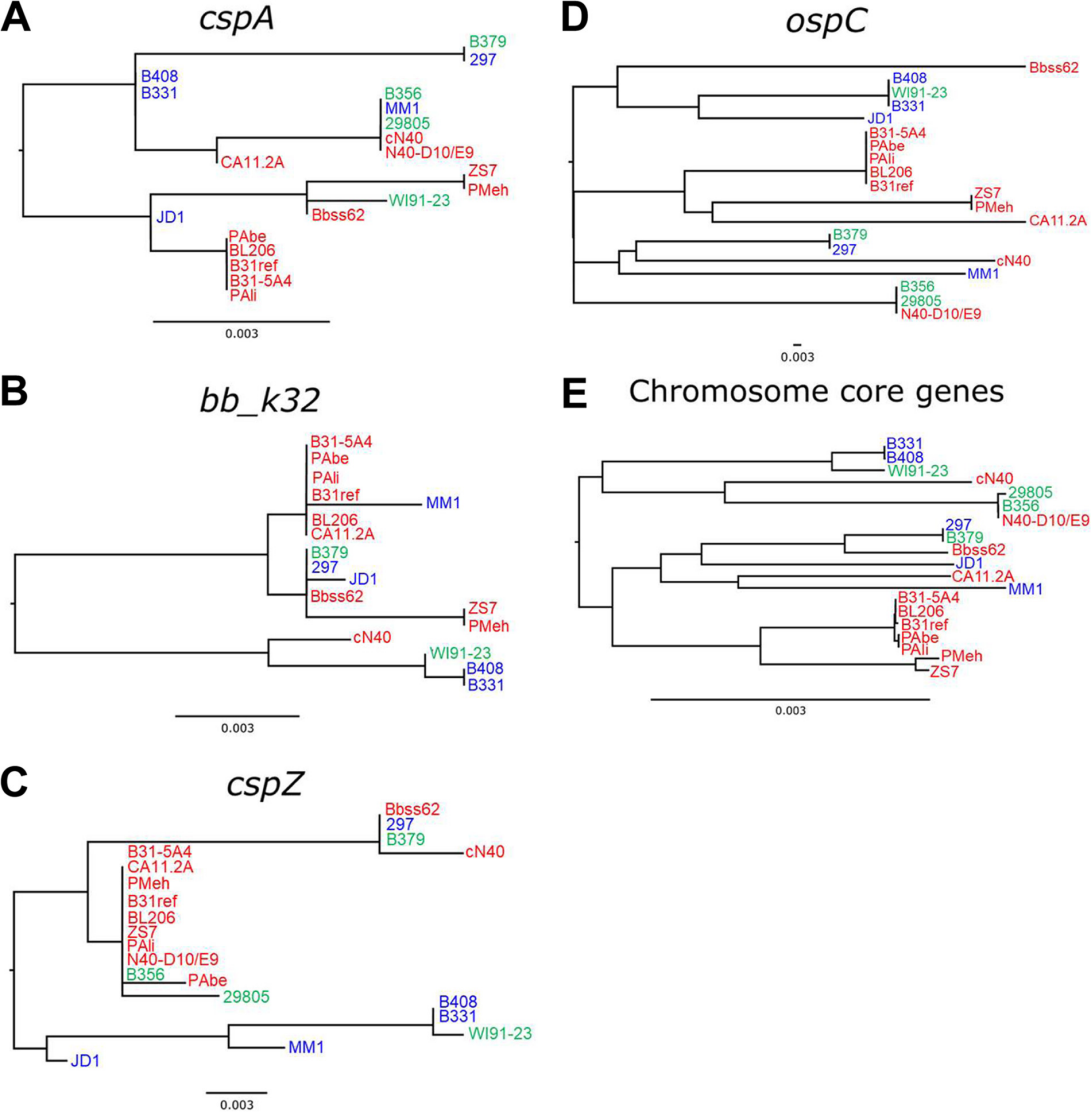
Phylogenetic trees of B. burgdorferi anti-complement proteins associate *ospC* and chromosomal core genes with host-specific complement inactivation activity. Individual phylogenies of *cspA* (A), *bb_k32* (B), *cspZ* (C), *ospC* (D), and chromosome core genes (E) represent evolutionary relationships among the 20 indicated B. burgdorferi strains with known complement evasion phenotypes. Labels are color-coded to represent strains that can efficiently inactivate complement from mouse (blue), quail (green), or both hosts (red). Strains that do not harbor particular loci are excluded from those respective trees.

**(ii) *ospC* evolution is associated with generalist and specialist anti-complement B. burgdorferi phenotypes.** We next investigated the phylogenetic relationships of the anti-complement protein loci from the above-mentioned B. burgdorferi strains. We reconstructed the phylogenetic history of the documented anti-complement genes (*cspA*, *bb_k32*, *ospC*, and *cspZ*) and the set of core chromosomal genes shared among all strains. *cspA* was the only Pfam54 gene included here, as no evidence supports that Pfam54 members other than CspA and its orthologs promote anti-complement activity (26, 29, 33–35). Although the proteins encoded by *ospE* and *elp* paralogs also display anti-complement phenotypes (25, 36–38), we did not include them because they are located on the highly homogenous family of 32-kilobase (kb) circular plasmids (cp32), which cause low-confidence assemblies derived from short-read sequencing (39). All the anti-complement and core chromosomal gene phylogenies exhibited paraphyletic groups of strains with divergent anti-complement activity phenotypes (Fig. 2). Across all trees, terminal groups often contained both mouse- and quail-specific anti-complement phenotypes (Fig. 2, strains with mouse- and quail-specific anti-complement phenotypes are presented in blue and green, respectively; strains with versatile anti-complement phenotypes are shown in red). The phylogenetic trees for *cspA*, *bb_k32*, and *cspZ* produced inconsistent correlations with Pagel’s λ distributions that ranged from 0 to 1 (Fig. 2A to C; Fig. S1A to C). The *ospC* phylogeny exhibited a consistent, strong signal of nonrandom trait evolution, and the anti-complement activity phenotype correlated was associated with tip position across the tree (λ = 0.88), suggesting an association between *ospC* genotype and host-specific anti-complement activity (Fig. 2D; Fig. S1D). The phylogeny produced from the core set of chromosomal genes also exhibited a consistent signal of correlation with anti-complement activity phenotype (λ = 0.93), suggesting that this phenotype may be driven by genome-wide evolution.

10.1128/msystems.00488-22.7 FIG S1 Distribution of Pagel’s λ values for 100 iterations for the correlation of the anti-complement gene phylogeny and the phenotypes of host-specific complement evasion. Histograms indicate the certainty of Pagel’s λ estimation for the analysis of host-specific anti-complement phenotype from Fig. 1 paired with the phylogeny of *cspA* (A), *bb_k32* (B), *cspZ* (C), *ospC* (D), and chromosome core genes (E). Only estimations for *ospC* and the core chromosomal genes result in stable estimations. Download FIG S1, JPG file, 0.6 MB. Copyright © 2022 Combs et al. 2022 Combs et al. https://creativecommons.org/licenses/by/4.0/ This content is distributed under the terms of the Creative Commons Attribution 4.0 International license.

### Strain-variable, host-specific, anti-complement phenotypes are linked to distinct levels of host-specific dissemination and genomic variation.

Our findings associated the host-specific, anti-complement phenotypes with the phylogeny of one anti-complement protein from various genotypes of B. burgdorferi strains. These results raise several intriguing questions: (i) Do genotypically distinct B. burgdorferi strains confer complement-dependent infectivity that varies among host types? (ii) If so, is genomic divergence associated with such an immunological variability of the phenotype of B. burgdorferi? To address these questions, we chose a strain from the group displaying either quail- or mouse-specific anti-complement phenotypes (B379 and B408, respectively) along with the strain with a versatile ability to inactivate complement from both host types (B31-5A4).

**FIG 3:**
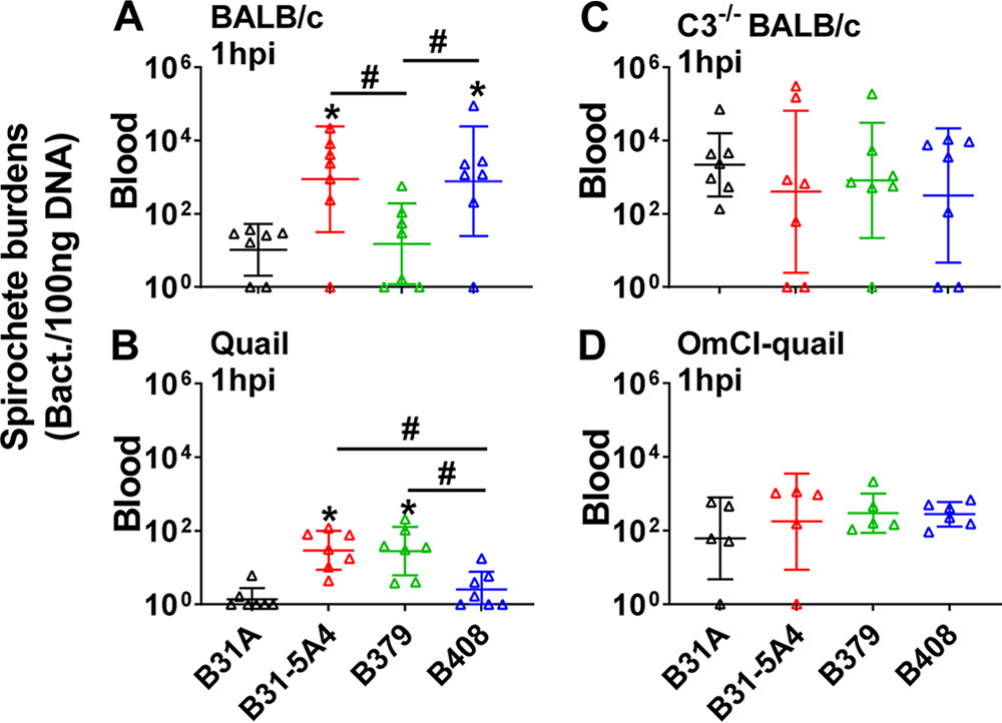
Complement dictates mouse and quail-specific short-term bloodstream survival of B. burgdorferi in a genotype-dependent manner. (A to D) BALB/c mice (A), PBS-treated quail (B), C3-deficient BALB/c mice (C3^−/−^BALB/c) (C), and *O. moubata* complement inhibitor [OmCI]-treated quail (D) were intravenously (i.v.) inoculated with B. burgdorferi strains B31-5A4, B379, or B408, or a high-passage, noninfectious B. burgdorferi strain B31A (control) (five animals/group for OmCI-treated quail and seven animals/groups for others). Blood was collected from these animals at 1 h post-inoculation (hpi), and bacterial burdens were quantified by quantitative PCR (qPCR). Shown are the geometric means of bacterial loads ± geometric standard deviations of five mice or quail per group. There were significant differences (*P* < 0.05, the Kruskal-Wallis test followed by the two-stage step-up method of Benjamini, Krieger, and Yekutieli) in the spirochete burdens from the burdens in B31A-infected animal blood (*) or between two strains relative to each other (#).

**(i) Genotypically distinct B. burgdorferi strains promote host-specific and complement-dependent phenotypes *in vivo*. (a) Strain-specific complement evasion activity promotes a host-specific variation in B. burgdorferi bloodstream survivability.** The presence of complement in host bloodstream raises the hypothesis of strain-specific anti-complement phenotypes conferring host-dependent B. burgdorferi bloodstream survivability. We thus examined the ability of strains B31-5A4, B379, and B408 to survive in the mouse or quail bloodstream 1-h postintravenous (i.v.) injection (hpi), a model established to test the short-term ability of B. burgdorferi to trigger bacteremia (31). In the blood from BALB/c mice, strains B31-5A4 and B408 yielded significantly greater levels of bacterial burdens than B379 and the negative-control strain, B31A (a high-passage strain that cannot efficiently survive in vertebrate bloodstream [31]) (Fig. 3A). Conversely, in quail blood, B31-5A4 and B379 induced significantly higher levels of bacterial loads than B408 and the control strain B31A (Fig. 3B). We observed indistinguishable burdens of these strains in the blood from C3^−/−^ BALB/c mice or OmCI-injected quail at 1 hpi (Fig. 3C and D). These results correlated the bloodstream survivability of B. burgdorferi strains with these strains’ anti-complement phenotypes in a host-specific manner.

**FIG 4:**
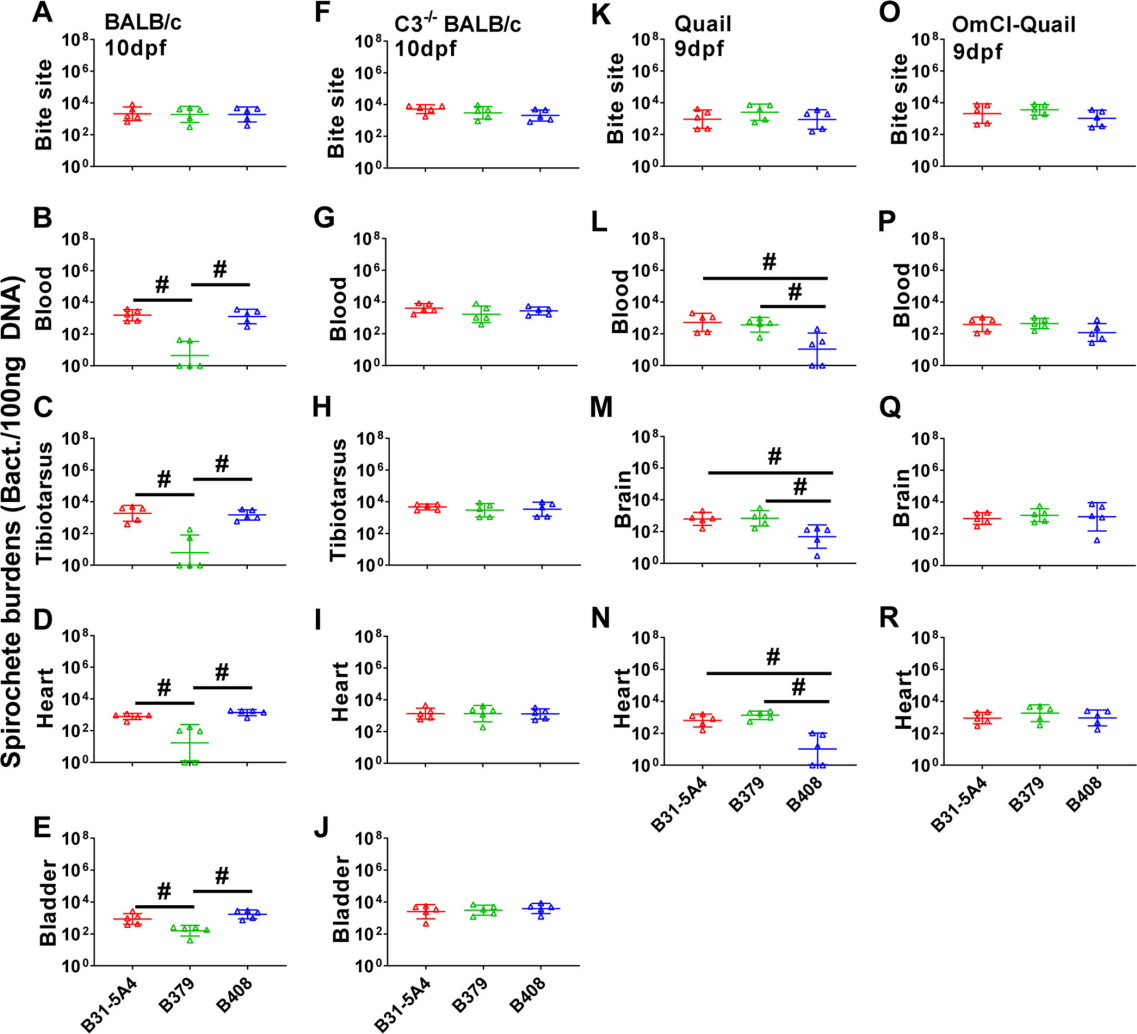
B. burgdorferi exhibits host- and bacterial genotype-specific early dissemination in a complement-dependent fashion. The I. scapularis nymphs carrying B. burgdorferi strains B31-5A4, B379, or B408 were allowed to feed until they were replete on five wild-type (A to E) or C3-deficient BALB/c (C3^−/−^ BALB/c) (F to J) mice or wild-type (K to N) or *O. moubata* complement inhibitor [OmCI]-treated (O to R) quail. The mice and quail were euthanized at 10 and 9 days after nymph feeding (dpf), respectively. The bacterial loads at the site where nymphs fed (bite site) (A, F), blood (B, G), tibiotarsus joints (C, H), heart (D, I), and bladder of mice (E, J) and the site of nymphs bite (bite site) (K, O), blood (L, P), brain (M, Q), and heart (N, R) of quail collected immediately after euthanasia were determined by quantitative PCR [qPCR]. The bacterial loads in tissues or blood were normalized to 100 ng total DNA. Shown are the geometric means of bacterial loads ± geometric standard deviation of five mice or quail per group. There were significant differences (*P* < 0.05, the Kruskal-Wallis test followed by the two-stage step-up method of Benjamini, Krieger, and Yekutieli) in the spirochete burdens between two strains relative to each other (#).

**(b) Complement is essential to determine the host-dependent, B. burgdorferi strain-specific early onset of dissemination.** The ability of bacterial survival in the blood has been associated with phenotypes of disseminated infection (24, 31, 40). We thus investigated this by generating Ixodes scapularis nymphs carrying indistinguishable levels of B31-5A4, B379, or B408 (Fig. S2A). After allowing these nymphs to feed on BALB/c mice, we evaluated bacterial burdens in replete nymphs and the tissues at 10 days post-tick feeding (dpf), the time point when spirochetes begin their systemic spread in mice, representing early onsets of dissemination (26) (Marcinkiewicz AL, and Lin YP Unpublished data.). We also measured bacterial burdens in the same fashion at 14 dpf, the later time point of dissemination to distal tissues. After tick feeding, we found similar levels of these strains in replete nymphs (Fig. S2B). At 10 dpf, B31-5A4, B379, and B408 displayed similar levels at the tick bite sites on skin (Fig. 4A), but the bacterial burdens of B379 were significantly lower than those of B31-5A4 or B408 in blood and all distal tissues (tibiotarsus joints, bladder, and heart; Fig. 4B to E). At 14 dpf, we found significantly lower bacterial burdens in all tissues, including the tick bite sites on the skin of B379-infected mice, compared to those from B31-5A4- or B408-infected mice (Fig. S3A to E). We also tested the role of complement in determining such a strain-specific dissemination by infecting the complement-deficient BALB/c mice (C3^−/−^ mice) in the same manner. Although bacterial burdens in all tissues from B379-infected mice remain significantly lower than those from B31-5A4- or B408-infected mice at 14 dpf (Fig. S3F to J), the infection of all tested strains yielded similar levels of bacterial loads in all tissues and replete nymphs at 10 dpf (Fig. 4F to J; Fig. S2D). These results indicate an essential role for complement in determining strain specificity at the beginning of dissemination.

10.1128/msystems.00488-22.8 FIG S2 The tested B. burgdorferi strains in this study exhibited similar burdens in flat and fed nymphs. C3-deficient BALB/c (C3^−/−^ BALB/c) mice were intradermally inoculated at the dose of 10^5^ spirochetes/mL BSK medium without rabbit sera with B. burgdorferi strains B31-5A4, B379, or B408. At 14 days after infection, the uninfected I. scapularis larval ticks were allowed to feed on each of these mice until they were replete. After the replete larvae molt into flat nymphs, those B. burgdorferi-infected flat nymphs were allowed to feed on BALB/c mice (B), PBS-treated quail (C), C3^−/−^ BALB/c mice (D), or *O. moubata* complement inhibitor [OmCI]-treated quail (E) to repletion. The bacterial loads in flat nymphs (A) or replate nymphs (B to E) were determined by quantitative PCR [qPCR]. Shown are the geometric mean ± geometric standard deviation of 8 flat nymphs or nymphs feeding on BALB/c mice, seven nymphs feeding on quail or C3^−/−^ BALB/c mice or 10 nymphs feeding on OmCI-treated quail (except for B408-infected nymphs feeding on OmCI-treated quail, there were 8 nymphs/group). There was no statistical difference (*P* > 0.05) of the spirochete burdens among different groups of the replete ticks using the Kruskal-Wallis test with the two-stage step-up method of Benjamini, Krieger, and Yekutieli. Download FIG S2, JPG file, 0.4 MB. Copyright © 2022 Combs et al. 2022 Combs et al. https://creativecommons.org/licenses/by/4.0/ This content is distributed under the terms of the Creative Commons Attribution 4.0 International license.

10.1128/msystems.00488-22.9 FIG S3 Complement is not sufficient to dictate B. burgdorferi genotype-specific bloodstream survival and tissue colonization in mice at 14 days postfeeding (dpf). The I. scapularis nymphs carrying B. burgdorferi strains B31-5A4, B379, or B408 were allowed to feed until they are replete on five BALB/c mice (A to E) or C3-deficient BALB/c (C3^−/−^ BALB/c) mice (F to J) in BALB/c background. The mice were euthanized at 14 dpf, respectively. The bacterial loads at the site where nymphs fed (bite site) (A, F), blood (B, G), tibiotarsus joints (C, H), bladder (D, I), and heart (E, J) of mice collected immediately after euthanasia were determined by quantitative PCR [qPCR]. The bacterial loads in tissues or blood were normalized to 100 ng total DNA. Shown are the geometric means of bacterial loads ± geometric standard deviation of five mice or quail per group. There were significant differences (*P* < 0.05, the Kruskal-Wallis test followed by the two-stage step-up method of Benjamini, Krieger, and Yekutieli) in the spirochete burdens between two strains relative to each other (#). Download FIG S3, JPG file, 0.7 MB. Copyright © 2022 Combs et al. 2022 Combs et al. https://creativecommons.org/licenses/by/4.0/ This content is distributed under the terms of the Creative Commons Attribution 4.0 International license.

We then tested whether such a strain-specific, complement-dependent infectious phenotype at the beginning of dissemination is dependent on the host used in the study. We allowed the nymphs carrying B31-5A4, B379, or B408 to feed on quail and measured bacterial burdens at 9 dpf, the time point when dissemination begins in quail, which represents the early infection onset for quail infected via tick feeding (26) (Marcinkiewicz AL, and Lin YP Unpublished data.). Quail-associated replete nymphs had similar levels of all tested strains, like mouse-associated replete nymphs (Fig. S2C). At 9 dpf, all strains exhibited indistinguishable levels of bacterial burdens at the tick bite sites on skin (Fig. 4K), similar to the infection in mice. However, unlike in mice, the B408 infection resulted in lower bacterial loads in quail blood and distal tissues (brain and heart) compared to B31-5A4 and B379 (Fig. 4L to N). Once those infected ticks fed on complement-deficient quail (OmCI-injected quail) for 9 days, we found indistinguishable levels of bacterial burdens in replete nymphs and any tested tissues (Fig. 4O to R; Fig. S2E). Together with the results from mice, these findings suggest an essential role of complement in dictating strain-specific, host-dependent early onsets of dissemination.

**FIG 5:**
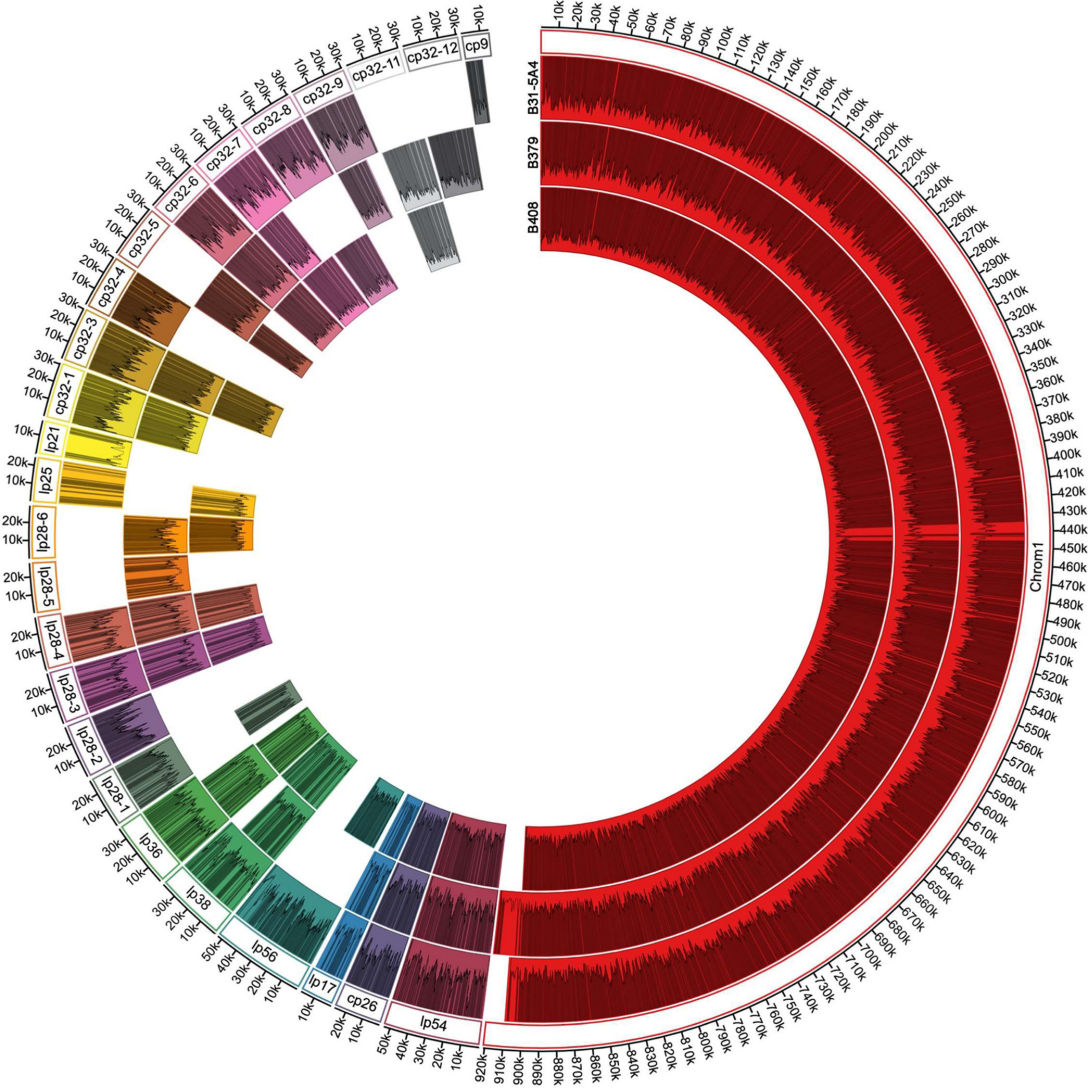
Genomic content of three B. burgdorferi strains. The genome of B. burgdorferi strains B31-5A4, B379, or B408 is represented by a concentric circle, and each color-coded individual segment represents a plasmid or the main chromosome, defined by labels provided in the outermost ring. Gaps in the ring represent plasmids that are not present in the genome at the time of sequencing. Gray lines within each segment represent gene annotations, and the plots within each segment represent the number of methylated nucleotides per 1,000 bases.

**(ii) Genomic divergence in B. burgdorferi strains with distinct host-specific anti-complement phenotypes.** The host- and strain-specific, complement-dependent infectivity of B31-5A4, B379, and B408 raises the likelihood of differentiation in genome content, organization, and patterns of epigenetic modifications in these strains. We generated high-quality genome sequences and assemblies of strains B31-5A4, B379, and B408 using a long-read approach (high-fidelity [HiFi] reads with circular consensus sequencing on the Pacific Biosciences platform) (Fig. 5). While each genome contained a single highly homologous linear chromosome, they each exhibited a unique suite and number of plasmids. B31-5A4 contains 11 linear and 9 circular plasmids. B379 contains 8 linear and 9 circular plasmids, although an additional plasmid (lp21) appears fused to the 3′ end of its linear chromosome. B408 contains 10 linear and 7 circular plasmids. Gene annotations revealed a variable gene content, with 1,527, 1,435, and 1,408 annotations identified in B31-5A4, B379, and B408, respectively (Table S1). We also identified methylation motifs, including two previously unreported motifs (GNAAGC in B379 and GAAGG in B408). The methylation rates of each strain vary, with B408 having the lowest rate among three strains (1.03 base methylations/1,000 bp) (Table S2).

10.1128/msystems.00488-22.1 TABLE S1 Long-read sequencing measures of B. burgdorferi strains B31-5A4, B379, and B408. Download Table S1, DOCX file, 0.02 MB. Copyright © 2022 Combs et al. 2022 Combs et al. https://creativecommons.org/licenses/by/4.0/ This content is distributed under the terms of the Creative Commons Attribution 4.0 International license.

10.1128/msystems.00488-22.2 TABLE S2 Methylation motifs identified in B31-5A4, B379, and B408. Download Table S2, DOCX file, 0.02 MB. Copyright © 2022 Combs et al. 2022 Combs et al. https://creativecommons.org/licenses/by/4.0/ This content is distributed under the terms of the Creative Commons Attribution 4.0 International license.

**FIG 6:**
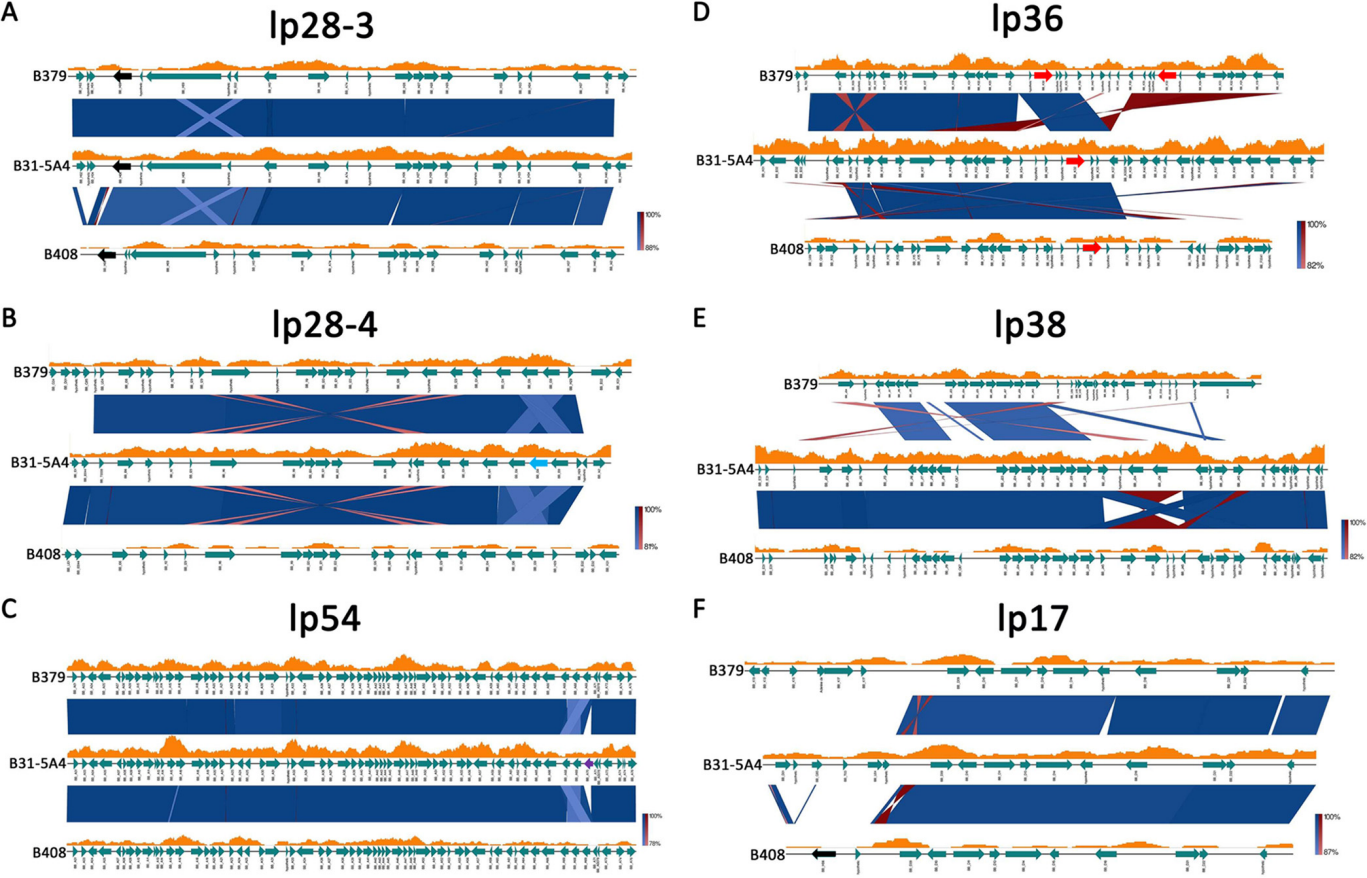
Genomic comparison reveals plasmid-specific differences among B. burgdorferi strains with distinct host-specific anti-complement phenotypes. The sequences of lp28-3 (A), lp28-4 (B), lp54 (C), lp36 (D), lp38 (E), and lp17 (F) from B. burgdorferi strains B31-5A4, B379, and B408 are represented by black lines, and gene annotations are depicted by teal arrows, labeled according to homology to known genes in the reference genome of B. burgdorferi strain B31. Segments connecting each strain represent filtered Basic Local Alignment Search Tool [BLAST] results in either the same orientation (blue) or opposite orientation (red), with darker shades representing closer matches. Orange graphs above each strain depict the sliding window calculation of methylated nucleotides per 1,000 bases. The colored arrows indicated the loci of *cspZ* (black), *bb_i38* (light blue), *bb_a70* (purple), and *bb_k32* (red).

**(a) Strain-specific duplication and deletion of loci including anti-complement genes.** Many regions of high similarity between B31-5A4, B379, and B408 emerged, including all across the linear chromosome, circular plasmid 26 (cp26) and much of lp28-3, lp28-4, and lp54 (Fig. 5 and 6A to C; Table S4). In contrast, we identified highly divergent gene content and organization along lp36. Both B379 and B408 exhibited an ~14-kb deletion at the 3′ end of lp36, compared with B31-5A4 (Fig. 6D). This deletion has removed an entire group of Pfam75 immunogenetic proteins, including P37 (i.e., BB_K50), which are not found elsewhere in the genome (Fig. 6D). Similarly, the lp38 of B379 retained only 12 of the 35 genes found on lp38 of B31-5A4 and B408. The latter two strains appear to have a highly conserved lp38 (Fig. 6E). Additionally, the 5′ end of lp17 exhibits high variability; in B379, this plasmid exhibits an ~4-kb fragment from lp36, whereas in B408, it contains a 2.6-kb fragment from lp28-3 (Fig. 6F). Among other non-cp32 plasmids, few were found in a single strain (lp28-2 and cp9 only in B31-5A4; lp28-5 only in B379), while others were recovered from only two strains (lp56, lp28-1, and lp25 only absent in B379; lp28-6 only absent in B31-5A4) (Fig. 5).

10.1128/msystems.00488-22.4 TABLE S4 E. coli strains and plasmids used in this study. Download Table S4, DOCX file, 0.03 MB. Copyright © 2022 Combs et al. 2022 Combs et al. https://creativecommons.org/licenses/by/4.0/ This content is distributed under the terms of the Creative Commons Attribution 4.0 International license.

We also investigated gene content of specific anti-complement loci (*ospC*, *bb_k32*, *cspZ*, and *cspA*) and their associated gene families in B31-5A4, B379, and B408. Each strain harbors a single copy of *ospC* on cp26 (red arrows in Fig. S4). While B31-5A4 and B408 have a single copy of *bb_k32* on lp36, B379 carries two copies on lp36 (red arrows in Fig. 6D). Additionally, whereas B31-5A4 and B379 each possesses one copy of *cspZ* on lp28-3, B408 contains two copies with a second *cspZ* locus found on the 5′ end of lp17 (black arrows in Fig. 6F). While we found *cspA* as a single gene copy in all three strains, we observed several deletions of other Pfam54 members. *bb_a70* was absent from B379 and B408 but found on lp54 in B31-5A4 (purple arrow in Fig. 6C). Further, *bb_i38* was found on lp28-4 in B31-5A4, although the neighboring Pfam54 loci of B379 and B408 were retained (light blue in Fig. 6B). Taken together, these results showcase regions of high plasmid similarity and variability, along with evidence of locus duplication and deletion events, including those specific to anti-complement activity, across B. burgdorferi strains with distinct host-specific anti-complement phenotypes.

10.1128/msystems.00488-22.10 FIG S4 No significant sequence variation, deletion, or duplication is found in cp26 among B31-5A4, B379, and B408. The sequence of each plasmid is represented by a black line, and gene annotations are depicted by teal arrows, labeled according to homology to known genes in B. burgdorferi strain B31. Segments connecting each strain represent filtered Basic Local Alignment Search Tool [BLAST] results in either the same orientation (blue) or opposite orientation (red), with darker shades representing closer matches. Orange graphs above each strain depict the sliding window calculation of methylated nucleotides per 1,000 bases. The red arrows indicate the loci of *ospC*. Download FIG S4, JPG file, 0.6 MB. Copyright © 2022 Combs et al. 2022 Combs et al. https://creativecommons.org/licenses/by/4.0/ This content is distributed under the terms of the Creative Commons Attribution 4.0 International license.

Across the 17 additional strains for which we tested serum survival phenotypes, variation in genome assembly quality and completeness prevent direct comparisons of plasmid organization but permit gene content analysis in association with strain-specific phenotypes. Our paired Roary and Scoary analysis identified only a single gene with known function, immunogenic P37, that was found in generalist strains but not within the genome of any host-adapted strains.

**FIG 7:**
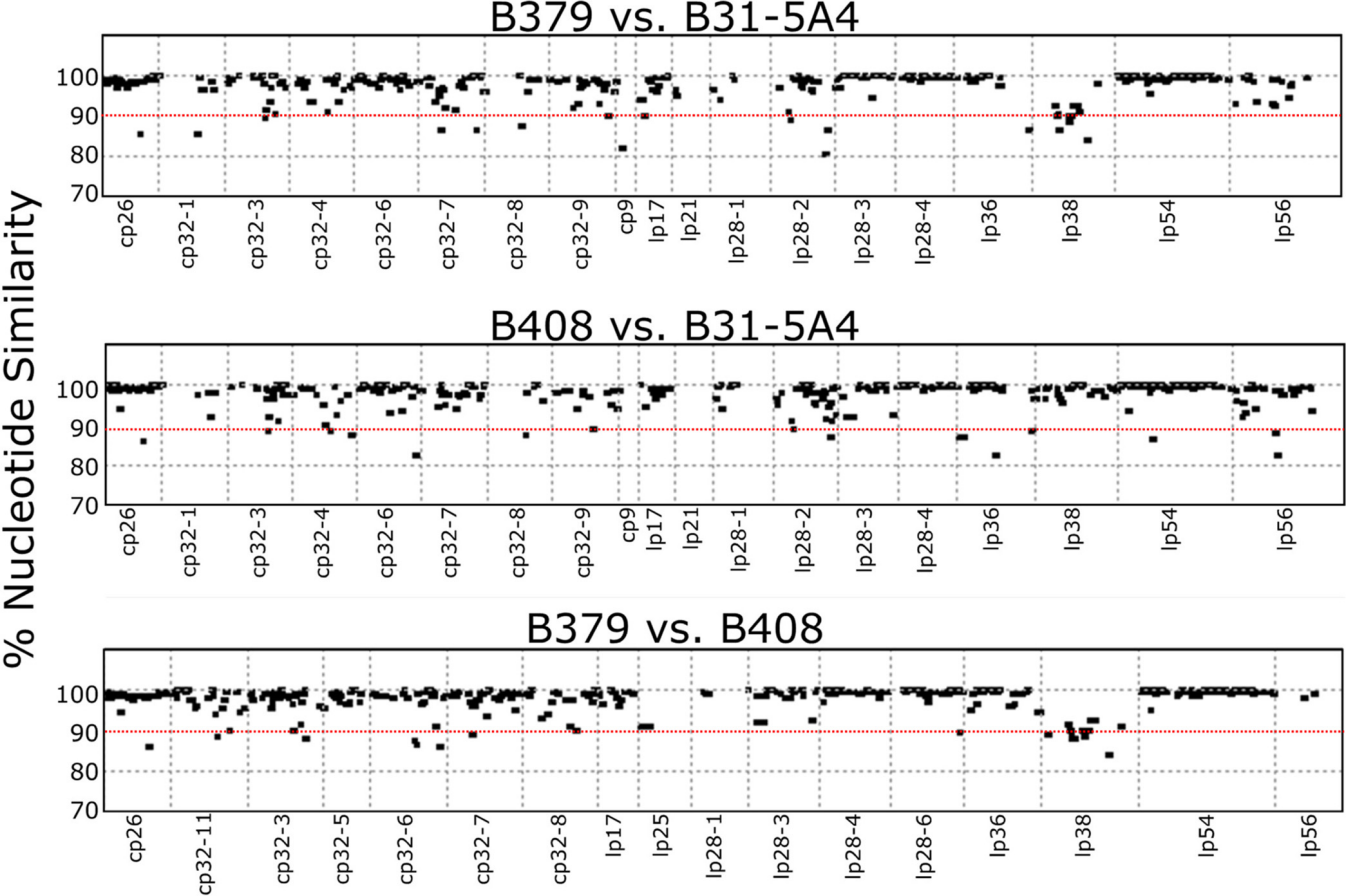
Loci-specific analysis identifies the polymorphic genes of the alleles among B. burgdorferi strains with distinct host-specific anti-complement phenotypes. Nucleotide similarity among annotated loci pairwise between B379 and B31-5A4 (top), B408 and B31-5A4 (middle), and B379 and B408 (bottom). The *x* axis represents the position of each annotation within the latter of the two compared genomes. The *y* axis represents the observed nucleotide similarity calculated using the NUCmer program. The red dotted lines indicate the threshold (90% nucleotide similarity) to define the polymorphic loci.

**(b) Characterization of variable genes with anti-complement determinants.** Most loci showed 90 to 100% similarity a high degree of conserved functions (Fig. 7). Few loci exhibited ≤90% similarity, such as the highly polymorphic *ospC* and *dbpA* (Table S3) (41, 42), and several genes documented to facilitate spirochete survival *in vitro* and *in vivo* and/or reduce infectivity after immunization, such as *bb_k13* and *vraA* (*bb_i16*) (Table S3) (43, 44). Among the genes encoding proteins with documented anti-complement phenotypes, *cspA*, *cspZ*, and *bb_k32* differ by relatively few polymorphisms, averaging 99.29, 96.91, and 97.24% similarity among all strain pairs, respectively, while the average similarity among *ospC* loci was 85.80% (Table S3).

10.1128/msystems.00488-22.3 TABLE S3 Variable genes from locus-specific comparison in B31-5A4, B379, and B408. Download Table S3, DOCX file, 0.03 MB. Copyright © 2022 Combs et al. 2022 Combs et al. https://creativecommons.org/licenses/by/4.0/ This content is distributed under the terms of the Creative Commons Attribution 4.0 International license.

## DISCUSSION

Differences in host association among pathogen species and strains are often the result of adaptive evolution by pathogens to host-specific immune responses (1, 45). The selective pressure imparted by host immune responses can result in the promotion of specific alleles or patterns of genomic variation in naturally occurring pathogens, known as multiple niche polymorphisms (MNPs) (5). Lyme borreliae exhibit strain-specific diversity in host range and genomic organization, with lipoprotein genes constituting a rapidly evolving part of the genome (4, 9, 11, 46, 47). Lipoproteins are associated with multiple cellular and immunological mechanisms influencing infection, thus making Lyme borreliae an ideal model for the investigation of the underlying mechanisms driving MNP-mediated host association. One such immunological mechanism is host-specific complement evasion, which has been shown to differ among Lyme borreliae species, whereby particular species are adapted to evade host-specific complement (14, 16, 48). In this study, our serum survivability and complement deposition assays demonstrated that B. burgdorferi strains exhibit either mouse-specific, quail-specific, or versatile complement evasion activity, extending the concept of host-specific complement evasion to different strains within one spirochete species.

Strains with quail-specific complement evasion disseminate more efficiently than other strains in wild-type quail during the early stages of dissemination (i.e., 9 dpf) but are indistinguishable from others in complement-deficient quail. Similarly, more efficient dissemination phenotypes in wild-type mice were conferred by the strains with mouse-specific complement evasion, compared to other strains, during the beginning of mouse dissemination (i.e., 10 dpf) but were indistinguishable from other strains in complement-deficient mice. These results are in agreement with the anti-complement activity documented to mediate early-onset-specific dissemination of B. burgdorferi (49–51). Therefore, our findings of strain-specific, host-dependent complement evasion activity and early-onset dissemination suggest that B. burgdorferi strains differ in their efficiency to disseminate, particularly at early stages, and such differences are host-specific and mediated by complement. Our findings also indicate that such a complement-dependent, strain-specific dissemination is less apparent at a later time point of spirochete dissemination (i.e., 14 dpf). In fact, additional mechanisms such as adaptive immune responses are involved in controlling spirochete colonization at later stages (13, 52). Such mechanisms may play more significant roles at later stages in diverse tissues, compared to complement. Thus, our results suggest the involvement of complement-independent mechanisms, impacting infection dynamics at tick bite sites on skin and distal tissues during later-onset (14 dpf) stages of infection, warranting further investigation.

We characterized genetic variation related to these divergent phenotypes using multiple approaches. First, we examined phylogeny-phenotype correlations at targeted loci with known roles in complement evasion using publicly available and new short-read-based genome assemblies of variable completeness. We examined gene content and variability, plasmid organization, and epigenomic modifications in strains representative of each complement evasion phenotype using high accuracy long-read sequencing. Among the examined complement evasion loci, only *ospC* gene genealogy correlated with host-specific anti-complement phenotypes among tested spirochete strains. Because associations between specific *ospC* types of B. burgdorferi strains and mammalian or avian reservoir animals have been observed (5, 53, 54), our results raise the possibility that OspC is an anti-complement determinant that directly contributes to host-specific complement evasion and infectivity. That possibility is consistent with our serum resistance assays using naive animal sera and OspC in binding C4b, interfering the impact of OspC-mediated evasion to an antibody-independent complement pathway (i.e., lectin pathway) (24). Additionally, this possibility is supported not only by the polymorphism of OspC among different B. burgdorferi strains but also by its complement ligand, C4b, which varies among different host taxa (i.e., ~35% sequence identity between mouse and quail) (55). However, such a possibility is at odds with several findings that B. burgdorferi strains carrying identical *ospC* sequences display distinct infectivity in mice (e.g., the strains N40-D10/E9 versus B356 [56, 57] or B379 from this study and 297 [58]). Thus, it is also possible that OspC serves as a marker, and other anti-complement loci linked to OspC in spirochetes contribute to such host-specific anti-complement phenotypes. Nonetheless, we did not find a correlation between host-specific complement evasion phenotypes and the phylogenies estimated for other tested anti-complement loci (i.e., *cspA*, *cspZ*, and *bb_k32*). These results suggest that other anti-complement determinants (e.g., *ospE* and *elp* paralogs not tested in this study) contribute to the complement evasion activity or that each of these variants contribute to the host-specific complement evasion to different extents (i.e., polygenic effects) in different strain-host-specific interactions. These possibilities are not mutually exclusive and would require future work to thoroughly examine.

The genomes of B. burgdorferi strains regularly recombine, and gene repertoires vary, leading to unique plasmid profiles (28, 59). Gene duplication and deletion have been consistently found in B. burgdorferi genomes, which may influence adaptive traits (60, 61). Consistent with these findings, we found evidence of recombination, duplication, and deletion in three representative B. burgdorferi strains with distinct host-specific phenotypes of hematogenous dissemination. These results suggest that the presence or absence of certain genes or the differences in copy numbers for those genes caused by genetic modification events affect distinct host-specific adaptation and eventually lead to B. burgdorferi diversification. For example, the members of Pfam75 are encoded on lp36 and expressed when spirochetes reside in hosts, although the roles of these members *in vivo* remain unclear (62, 63). We found that the strains that efficiently disseminate in a single host in this study (i.e., B379 and B408) share a large 3′ end deletion of lp36, resulting in the absence of Pfam75 genes, whereas the strain that efficiently spreads in multiple hosts (i.e., B31-5A4) maintains intact Pfam75. This is consistent with the genome of B. garinii, a species specifically adapted to birds that lacks many lp36-encoded loci, including Pfam75 genes (64). Our findings indicate the need for future studies to examine the role of these genes in determining host-specific infectivity.

When anti-complement genes were specifically examined in this comparative genomic study, we found that strains B379 and B408, with distinct host-specific dissemination phenotypes, exhibit duplication of different anti-complement genes (i.e., duplication of c*spZ* and *bb_k32* in B379 and B408, respectively). Gene duplication has been shown to confer a selective advantage by increasing gene expression via the dosage effect (61, 65). Our results thus raise the intriguing possibility that although specific genes across B. burgdorferi strains may exhibit high sequence similarity and confer similar host-specific infection phenotypes (i.e., complement evasion), dosage effects imparted by variable gene copy numbers may help determine the overall host-specific phenotypes. In fact, Lyme borreliae genomes are known to harbor numerous gene families, suggesting that gene duplications are a common strategy for adapting to selective pressure and improving adaptive potential (9, 28). A future study would investigate gene dosage effects and dosage compensation by deploying transcriptomics at different time points of infection. Further, our study added to the known diversity of methylation motifs involved in restriction/modification systems across B. burgdorferi strains that likely impact recombination across plasmids and/or differential gene expression (66–68). This supports the notion that the phenotypic variation among Lyme borreliae species or strains could also be driven by epigenetic differences in spirochetes. An examination of the expression differences among strains or the impact of horizontal gene transfer governed by restriction/modification systems would be essential for identifying epigenetic determinants of host association.

Our laboratory animal models and natural reservoir hosts are evolutionarily distant (house mice versus white-footed mice diverged 24 million years ago [mya]; quail and passerines [e.g., American robins] diverged 85 mya [69, 70]). This reflects the differences of spirochete-related phenotypes documented *in vitro* and *in vivo* between laboratory or reservoir hosts and the need to extend such a host association study to reservoir animals in a future work (e.g., house mice versus white-footed mice) (11, 71, 72). Additionally, these rodent and avian models were singularly infected with B. burgdorferi strains, including *ospC* type K (B379) and the *ospC* type I (B408) that are preferentially disseminated in quail and mice in this study, respectively. Such a singular infection may not address the coexistence of different strains found in ticks and vertebrate hosts in nature. However, there is no evidence to suggest that these strains coexist in ticks and hosts in nature in a consistent, host-dependent fashion. Finally, accurate assembly from short-read sequencing data of Lyme borreliae is challenging, as the genome of these species/strains include a large number of linear and circular plasmids, some of which are very conserved (cp32s) and/or constantly recombine (39, 59). These characteristics highlight the importance of the multiple polygenic traits in impacting multiple phenotypes (e.g., host-specific complement evasion and dissemination), which requires a comparative genomics approach, despite the fact that several single- and multilocus typing schemes have historically been used to characterize genotypic variation (intergenic spacer [IGS], RST, and MLST [9, 73]). Here, we applied a multidisciplinary approach to examine the concept of complement-mediated, host-specific infection, demonstrating the diversification of Lyme borreliae strains within single species. Comparing locus-specific phylogenetic versus phenotypic differences, combined with comparative genomics, enables us to identify the potential determinants of host association. Such results can provide the evolutionary framing of the Lyme disease system, paving the road in dissecting the molecular mechanisms of host-pathogen interactions.

## MATERIALS AND METHODS

### Ethics statement.

All mouse and quail experiments were performed in strict accordance with all provisions of the Animal Welfare Act, the Guide for the Care and Use of Laboratory Animals, and the Public Health Service (PHS) Policy on Humane Care and Use of Laboratory Animals. The protocol was approved by the Institutional Animal Care and Use Committee (IACUC) of Wadsworth Center, New York State Department of Health (protocol docket number 19-451). All efforts were made to minimize animal suffering.

### Mouse, quail, tick, bacterial strains, animal sera, and OmCI.

BALB/c and Swiss Webster mice were purchased from Taconic (Hudson, NY). C3^−/−^ mice in BALB/c background were generated from the C3^−/−^ (C57BL/6) purchased from The Jackson Laboratory (Bar Harbor, ME) as described in our previous study (29). Common quail (C. coturnix) were purchased from Cavendish Game Birds Farm (Springfield, VT). I. scapularis tick larvae were purchased from the National Tick Research and Education Center, Oklahoma State University (Stillwater, OK) or from BEI Resources (Manassas, VA). Lyme borreliae-infected nymphs were generated as described in the section “Generation of infected ticks.” The *Borrelia* and Escherichia coli strains used in this study are described in Table 1 and Table S4. E. coli strains Rosetta Origami (DE3) (MilliporeSigma, Burlington, MA), and derivatives were grown in Luria-Bertani (BD Bioscience) broth or agar, supplemented with kanamycin (50 μg/mL) or no antibiotics as appropriate. All B. burgdorferi strains were grown in BSK-II completed medium with no antibiotics and verified for their plasmid profiles for the strains that have the protocol established (74) prior to the experiments and/or maintained in the passage less than 10 to avoid the confounding factors caused by plasmid missing. The sera from white-footed mice were obtained as described previously (11), and quail sera were obtained from Canola Live Poultry Market (Brooklyn, NY). Prior to being used, these sera were screened with the C6 Lyme enzyme-linked immunosorbent assay (ELISA) kit (Diamedix, Miami Lakes, FL) to determine whether the individual from which it was collected had prior exposure to B. burgdorferi by detecting antibodies against the C6 peptide of the B. burgdorferi protein VlsE (75).

### Generation of recombinant OmCI and quail C8γ and antisera against quail C8γ.

To generate the plasmid in producing soluble OmCI proteins, a bicistronic expression plasmid was constructed to allow simultaneous production of the OmCI (NCBI Reference Sequence; AY560803.1; amino acids 19 to 168) and with mature human protein disulfide-isomerase (GenBank; X05130.1; amino acids 18 to 508) as described (76). The DNA fragments in this plasmid were first synthesized to include (i) the region of 67 to 518 nucleotides encoding OmCI (O. moubata complement inhibitor precursor), (ii) a linker and a ribosome-binding site (GGAGGCAAAAA), (iii) a translational starting site (ATGAAA), and (iv) the mature human protein disulfide-isomerase from the five to three prime ends (Synbio Technology, Monmouth Junction, NJ). That DNA fragment was then added the restriction enzyme sites, BamHI and SalI, at the 5′ and 3′ ends using PCR followed by being ligated into the vector, pET28a (MilliporeSigma), previously digested by the respective restriction enzymes. The resulting plasmid was then transformed into the E. coli strain Rossetta Origami (DE3). To generate the plasmid in producing the soluble γ chain of quail C8 (NCBI RefSeq XM_015878802.2; amino acid positions 50 to 228), the region of 300 to 839 nucleotides encoding *c8g* (γ chain of C8 from common quail) was synthesized (Synbio Technology) and cloned in the same fashion. The resulting plasmid was also transformed into the E. coli strain Rossetta Origami (DE3). The histidine-tagged quail C8γ and OmCI were produced and purified by nickel-nitrilotriacetic acid (Ni-NTA) affinity chromatography according to the manufacturer’s instructions (GE Healthcare, Piscataway, NJ). Antisera against quail C8γ were generated by immunizing 4-week-old Swiss Webster mice with the recombinant quail C8γ as described (77).

### Flow cytometry.

The determination of mouse C5b-9 or quail C8 deposition using fluorescence-activated cell sorting (FACS) was described previously with modifications (29, 30). In brief, phosphate-buffered saline (PBS) was used to wash spirochetes (1 × 10^8^ cells), which were then resuspended in the same buffer. The sera from mouse or quail were subsequently incubated with suspended spirochetes at a final concentration as 20% at 25°C for 1 h. After incubation, spirochetes were washed by PBS and then resuspended in HBSC-DB (25 mM HEPES acid, 150 mM NaCl, 1 mM MnCl_2_, 1 mM MgCl_2_, 0.25 mM CaCl_2_, 0.1% glucose, and 0.2% bovine serum albumin [BSA]). A rabbit anti-mouse C5b-9 polyclonal IgG (1:250×) (Complement Technology, Tyler, TX) and a mouse anti-quail C8 polyclonal serum (1:250×) generated in the section “Generation of recombinant OmCI and quail C8γ and antisera against quail C8γ” were used as primary antibody. An Alexa 647-conjugated goat anti-rabbit (ThermoFisher) or a goat anti-mouse IgG (ThermoFisher) (1:250×) was used as the secondary antibody. After staining, formalin (0.1%) was then added for fixing. The resulting fluorescence intensity of spirochetes was measured and analyzed by flow cytometry using a FACSCalibur (BD Bioscience) as described in the previous studies (30).

### Serum resistance assays.

The serum resistance of B. burgdorferi was measured as previously described, with modifications (11, 29, 30). To determine the survivability of each of the B. burgdorferi strains in the sera, the mid-log phase of each of these strains was cultivated in triplicate and diluted to a final concentration of 5 × 10^6^ bacteria/mL into BSK-II medium without rabbit sera. The high-passage, noninfectious, and serum-sensitive B. burgdorferi strain B313 was also included as control. The cell suspensions were mixed with sera collected from naive white-footed mice or quail (60% [vol/vol] spirochetes and 40% [vol/vol] sera) in the presence or absence of 2 μM cobra venom factor (CVF) (Complement Technology) or recombinant OmCI. The sera preincubated at 65°C for 2 h (heat-inactivated sera) were included as a control. To determine the impact of OmCI treatment to reduce the complement-mediated killing activity of quail, the quail were subcutaneously injected with OmCI (1 mg/kg of quail) or PBS buffer (control), and the sera were collected at 9 days postinjection (dpi). B. burgdorferi strain B313 was cultivated and then mixed with those quail sera in the same fashion indicated above. The bacteria mixed with heat-inactivated serum samples were also included as a control. We determined the survivability of bacteria by counting the number of motile and immotile bacteria, in which we have shown to yield similar results to the methodology counting the bacteria based on live: dead staining (30, 78). Basically, the number of motile spirochetes was measured under dark field microscopy at 0 and 4 h following incubation with sera. The percentage of survival of B. burgdorferi was calculated by the normalization of motile spirochetes at 4 h postincubation to that immediately after incubation with sera.

### Evolutionary genomics.

To evaluate the correlation between phylogenetic signal and serum survivability phenotypes, we compiled genomic sequences for each of the 20 B. burgdorferi strains, as well as for the Borrelia bissettiae strain DN127 (Table 1). Of these, fully or partially assembled genomes of 11 strains were available from GenBank: JD1, MM1, CA11.2A, ZS7, PAli, PAbe, B31, cN40, B331, WI91-23, and 29805 (accession numbers shown in Table 1). One strain, PMeh, was unassembled but represented by raw Illumina reads on the Sequence Read Archive (SRA) (Table 1). Genomic DNA for four strains, B356, N40-D10/E9, 297, and Bbss62, was extracted from cultures. Library preparation was carried out with the Nextera DNA Flex library preparation kit (Illumina, San Diego, CA) and sequenced on a MiSeq instrument using the v2 500-cycle kit at the Advanced Genomic Technologies Cluster, Wadsworth Center (Albany, NY). Raw reads for PMeh, B356, N40-D10/E9, 297, and Bbss62 were quality-filtered and trimmed using the *trim_galore* wrapper script specifying a minimum quality score of 20 and a minimum length of 60 nucleotides (https://www.bioinformatics.babraham.ac.uk/projects/trim_galore). Assembly took place in SPAdes v3.15.3 using the “–isolate” mode (79). Sequencing and assembly details for B31-5A4, B408, and B379 are described below.

The linear chromosome of B. burgdorferi contains genes essential for biological functions and is subjected to strong purifying selection, providing a useful representation of neutral population genetic relationships among strains (80). We extracted core genes found across each of the 20 B. burgdorferi genomes and the Borrelia bissettii strain DN127 using Roary v3.13.0 (parameters: –i 0.9, –e, –s), after annotation with PROKKA v1.14.6 (Seemann 2014). We used IQTree v1.6.12 to estimate a phylogenetic tree from the core set of chromosomal genes, partitioning by gene, implementing a full substitution model testing procedure, along with the ultrafast bootstrap procedure and the Shimodaira-Hasegawa-like approximate likelihood ratio test to gauge internode branch support, using the following parameters: –bb 10000 –alrt 10000 –safe –p –t RANDOM (81).

We reconstructed the phylogenetic history of several genes known to be involved in host complement evasion by B. burgdorferi, including *ospC*, *cspZ*, *cspA*, and *bb_k32*. We noted that the strains 29805, N40-D10/E9, and B356 are missing *bb_k32* alleles and thus did not include those alleles in the *bb_k32*-derived phylogeny. Sequences for each of these genes were aligned as translated amino acids using MAFFT v7.480 (82), and phylogenies were constructed using IQTree v1.6.12 and the same parameters listed above. We calculated Pagel’s λ, a measure of phylogenetic signal (83), specifying phenotype as a discrete trait, using the *geiger* R package (84). To evaluate analytical consistency, we ran each analysis 100 times and built a distribution of resulting λ values.

### Generation of infected ticks.

Generating infected I. scapularis ticks has been described previously with modifications (85). BALB/c C3-deficient mice were infected intradermally with 10^5^ of each of the B. burgdorferi strains. The DNA extracted from the ear tissues was applied to quantitative PCR (qPCR) to verify DNA positivity of B. burgdorferi as verification of infection (see section “Quantification of spirochete burden”). The uninfected Ixodes scapularis larvae (~100 to 200 larvae/mouse) were allowed to feed to repletion on the infected mice as described previously (26). The engorged larvae were collected and permitted to molt into nymphs in a desiccator at room temperature with 95% relative humidity and light dark control (light to dark, 16:8 h).

### Intravenous inoculation of B. burgdorferi.

The short-term intravenous inoculation experiments were performed, as described with modifications (24, 31). The 4- to 6-week-old male and female BALB/c or C3^−/−^ mice in BALB/c background were inoculated with 1 × 10^7^ B. burgdorferi cells in 100 μL via lateral tail veins, whereas 4- to 6-week-old male and female PBS or OmCI-treated quail were injected with 1 × 10^8^ B. burgdorferi cells in 100 μL via brachial veins in the wing. At 1 h after inoculation, the mice were euthanized to collect blood by cardiac puncture, while the blood from quail was collected from the brachial vein from the other side of wing. DNA isolated from the blood samples was used for quantitation of Borrelia by qPCR described below (24, 31).

### Mouse and quail infection by ticks.

The flat nymphs were placed in a chamber on 4- to 6-week-old male and female BALB/c or C3^−/−^ mice in BALB/c background or 4- to 6-week-old male and female PBS- or OmCI-treated-quail as described previously (26). For OmCI-treated quail, the quail were subcutaneously injected with OmCI (1 mg/kg of quail) a day prior to the nymph feeding. The engorged nymphs were obtained from the chambers at 4 days after tick feeding. Blood, tick placement site of skin, and heart from the quail and mice, bladder and tibiotarsus joints from the mice, and brain from the quail were collected at 9 (for quail), 10, or 14 (for mice) days after nymph feeding.

### Quantification of spirochete burden.

The DNA from tissues, blood, or ticks was extracted as described previously (26). qPCR was then performed to quantitate bacterial loads. Spirochete genomic equivalents were calculated using an ABI 7500 real-time PCR system (ThermoFisher Scientific) in conjunction with PowerUp SYBR green Master Mix (ThermoFisher Scientific) based on amplification of the Lyme borreliae 16S rRNA gene using primers BB16srRNAfp and BB16srRNArp (Table S5) with the amplification cycle as described previously (30). The number of 16srRNA copies was calculated by establishing a threshold cycle (*C_q_*) standard curve of a known number of 16srRNA gene extracted from B. burgdorferi strain B31-5A4 and then comparing the Cq values of the experimental samples.

10.1128/msystems.00488-22.5 TABLE S5 Primers used in this study. Download Table S5, DOCX file, 0.03 MB. Copyright © 2022 Combs et al. 2022 Combs et al. https://creativecommons.org/licenses/by/4.0/ This content is distributed under the terms of the Creative Commons Attribution 4.0 International license.

### Comparative genomics.

**Sequencing and assembly.** B31-5A4, B379, and B408 were grown for genomic sequencing as described above, and genomic DNA was extracted using a cetyltrimethylammonium bromide and organic extraction method (86). The genomic DNA was assessed for shearing with gel electrophoresis. Genomic libraries were independently prepared for sequencing using the SMRTbell library prep kit, with a mean sheared genomic DNA distribution in the range of 4 to 6 kb and then sequenced on a PacBio RSII system at the Icahn School of Medicine at Mount Sinai Genomics Core Facility (New York, NY). Genomes were assembled using the default HGAP3 pipeline. Upon inspection, all three assembled genomes revealed evidence of “adapter skipping” artifacts in the form of long (5 to 20 kb) inverted repeats found only at the termini of many linear plasmids, a known issue commonly identified across Borrelia genomes (9). Adapter skipping occurs when one hairpin adapter fails to ligate during library preparation, causing individual subreads to contain both sense and antisense sequences (personal communication with PacBio field applications scientist, Dan Browne, over email, beginning in March 2021). While the *recalladapters* script is now available to correct this issue, it is not compatible with RSII system-derived sequences. We therefore regrew the cultures and prepared genomic DNA for each strain as described above and sequenced each on a Sequel I instrument. Initial assembly with HGAP4 using default parameters revealed nearly identical adapter skipping artifacts, which were ameliorated only after implementing custom subread filtering scripts provided by PacBio’s software development team (Text S1).

10.1128/msystems.00488-22.6 TEXT S1 Scripts used for additional subread filtering to remove adapter skipping reads from subread data set, provided by Pacific Biosciences product application specialists and developer team. Download Text S1, DOCX file, 0.02 MB. Copyright © 2022 Combs et al. 2022 Combs et al. https://creativecommons.org/licenses/by/4.0/ This content is distributed under the terms of the Creative Commons Attribution 4.0 International license.

HiFi reads were next generated subread consensus sequence for each strain using PacBio’s *ccs* script (github.com/PacificBiosciences/pbbioconda). Genomes for each strain were assembled using Canu v2.1 using the following parameters: –pacbio-hifi, minReadLength = 1,500, minOverlapLength = 1,500 (87). Circular contigs identified by Canu were trimmed and rotated to match the start position of reference sequences B31 or of JD1 and cN40 for plasmids not found in B31, using Gepard v1.40 and the *fasta_shift* script available in the fasta_tools package (github.com/b-brankovics/fasta_tools). The plasmids were identified by homology comparisons to known PFam32 loci, plasmid partitioning genes commonly used to identify Borrelia plasmids (60) and based on Gepard v1.40 dotplot and BLASTn matches to known plasmids from B31, JD1, and N40 reference genomes. Genomes were annotated with PROKKA v1.14.6 (Seemann 2014) using proteins from the B31 reference genome for initial comparison (accession number GCA_000008685.2).

**Base modifications.** For each genome, base modifications and motifs were detected using Pacific Biosciences SMRT analysis pipeline, in which modification calls were retained only if they had a quality value of 400 or greater for B31-5A4 and B379 or 200 or greater for B408. Quality value thresholds were determined from breaks observed in the quality value distributions, as suggested by Pacific Biosciences Product Application Specialists. The positions of methylated motifs were extracted, and a 1,000-bp sliding window was used to visualize and compare genome-wide methylation rates using the *zoo* R package (88).

**Plasmid and gene comparison.** Shared plasmids between B31-5A4, B379, and B408 were compared using BLAST sequence alignments, gene annotations, and the number of methylated m6A motifs per 1,000 bp with Easyfig (89). To identify nucleotide similarity among annotated genes, we used NUCmer and mummerplot (using parameter –c for percent identity plots) from the MUMmer v3.1 package (90).

### Statistical analysis.

Significant differences between samples were assessed using the Mann-Whitney *U* test or the Kruskal-Wallis test with the two-stage step-up method of Benjamini, Krieger, and Yekutieli. A *P* value < 0.05 (*) or (#) was considered to be significant (91).

### Data availability.

All programs, versions, and parameters used in this study are described in the Materials and Methods section. The GenBank accession codes used are listed in Table 1.
