# Supplementary figures and images for: Phylogenomic Diversity Elucidates Mechanistic Insights into Lyme Borreliae-Host Association
Source: mSystems. 2022 Aug 8;7(4):e00488-22. doi: 10.1128/msystems.00488-22 (PMC9426539; doi:10.1128/msystems.00488-22)

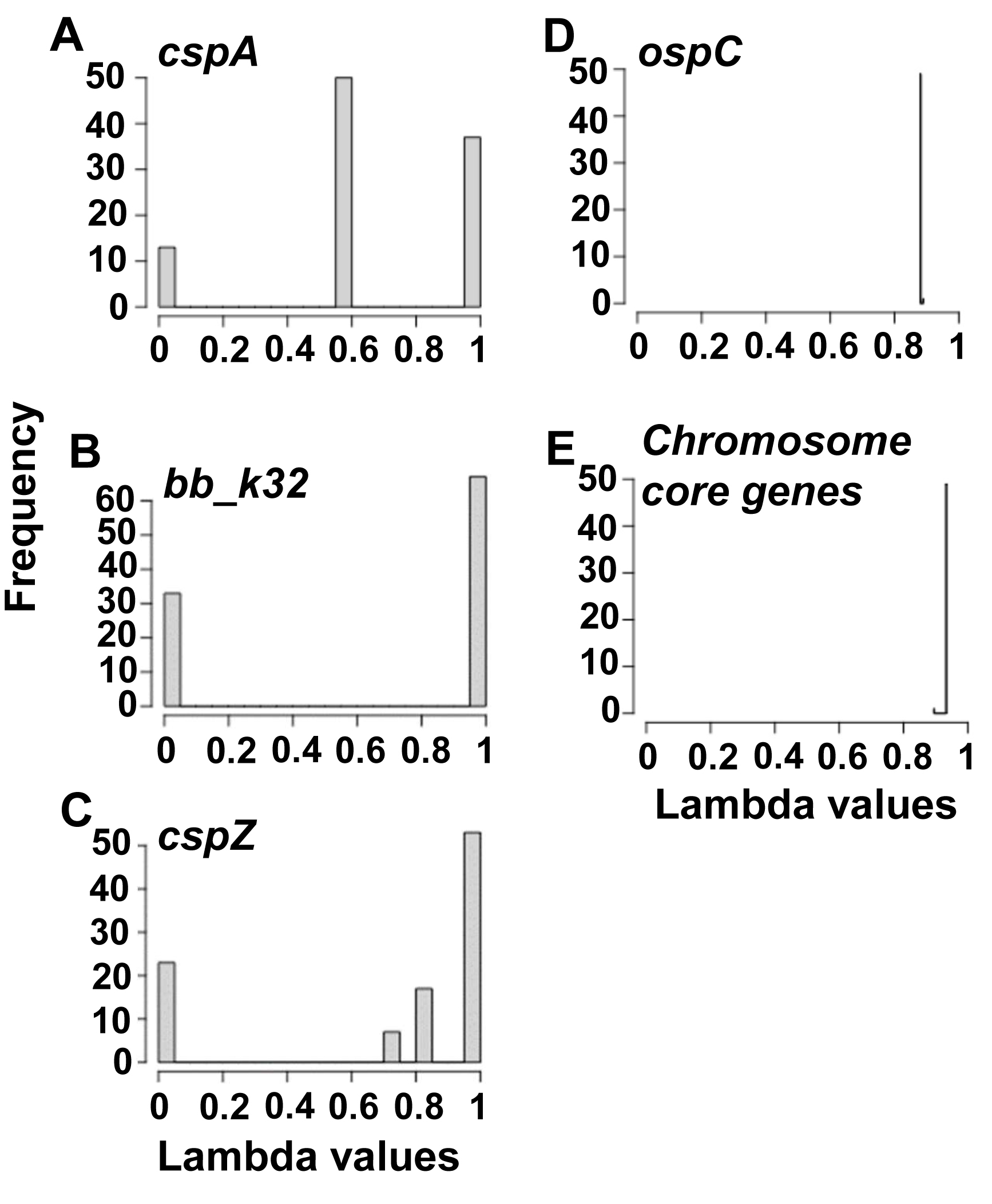

Supplement: FIG S1 [file msystems.00488-22-s0007.jpg]

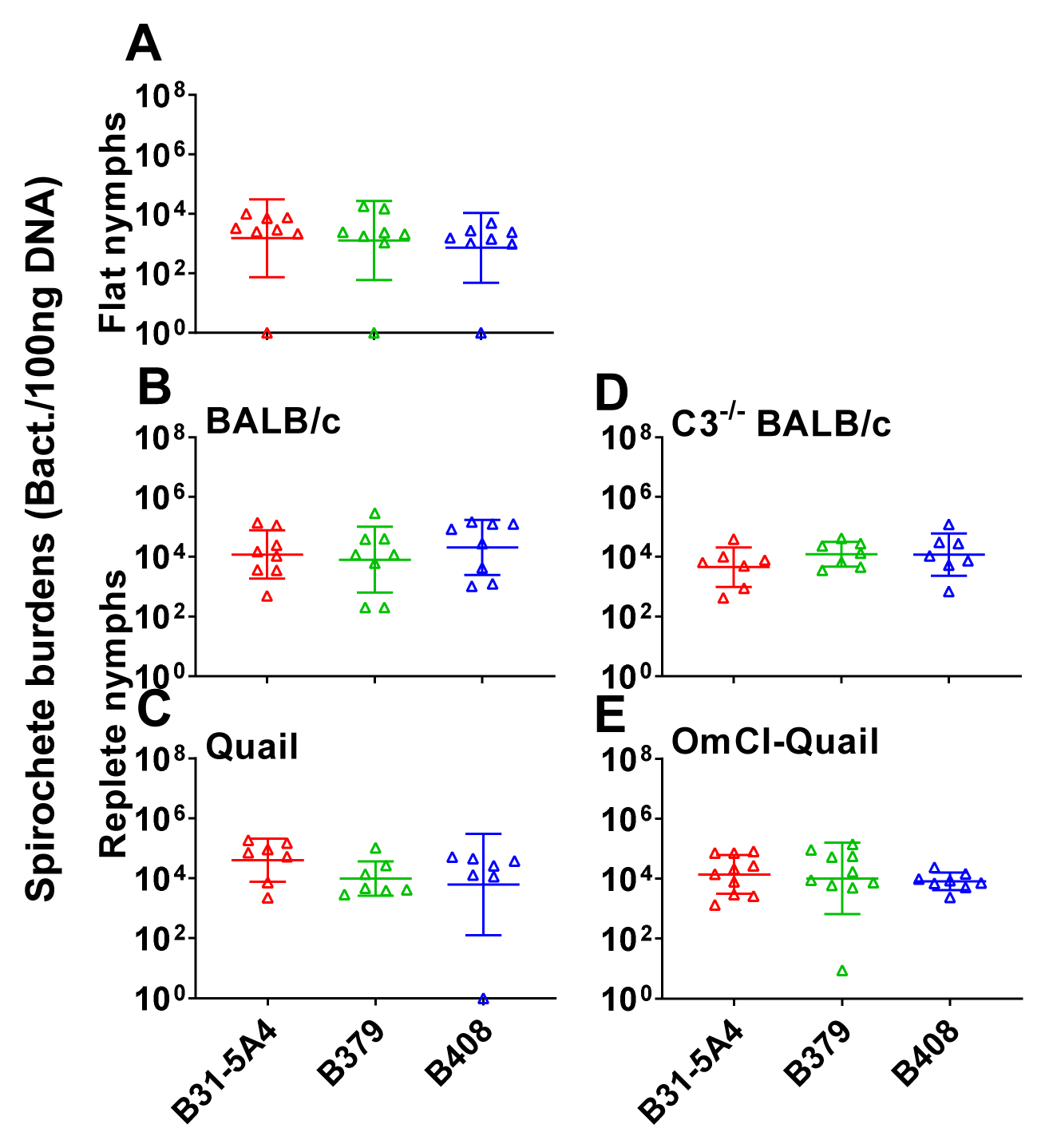

Supplement: FIG S2 [file msystems.00488-22-s0008.jpg]

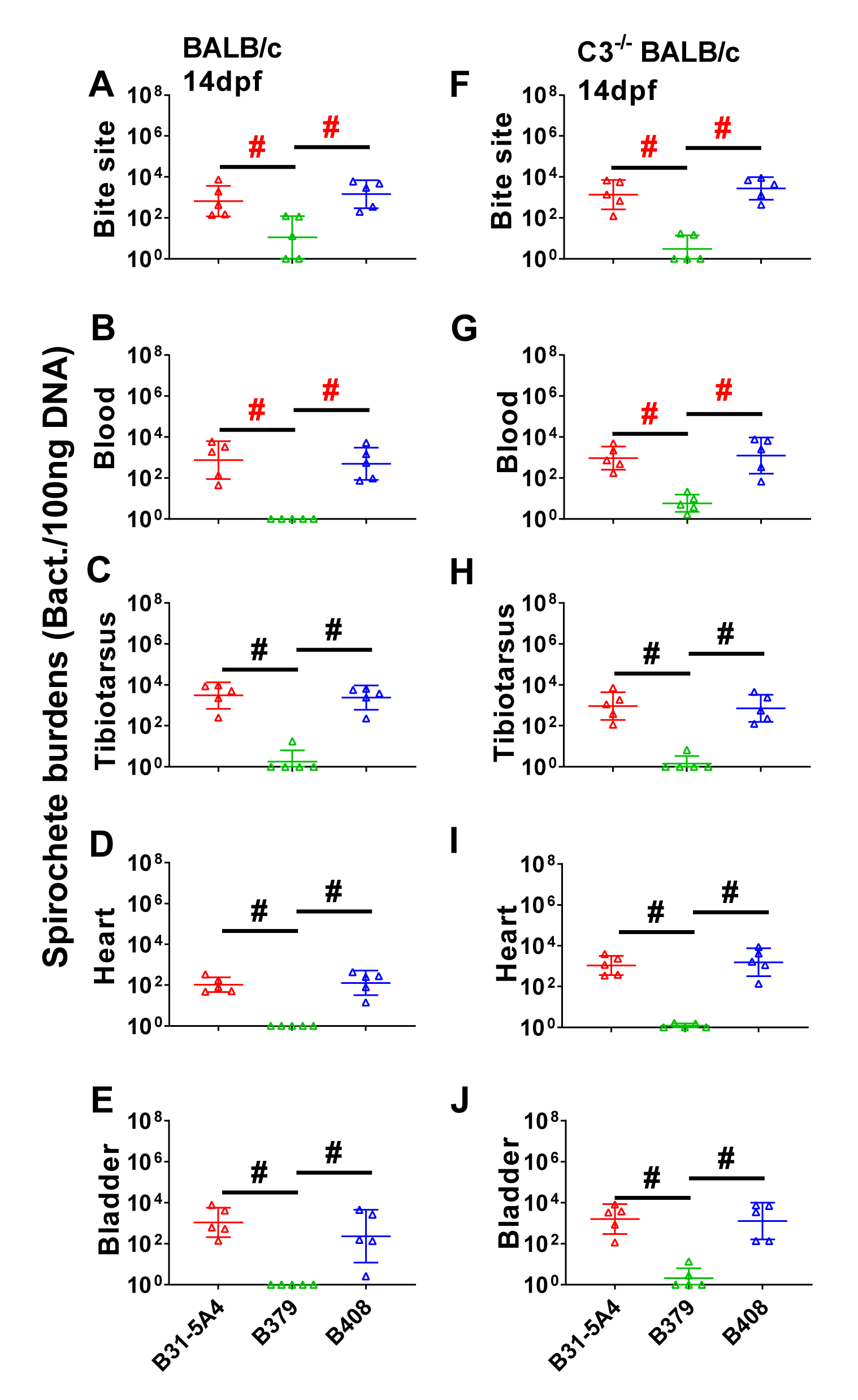

Supplement: FIG S3 [file msystems.00488-22-s0009.jpg]

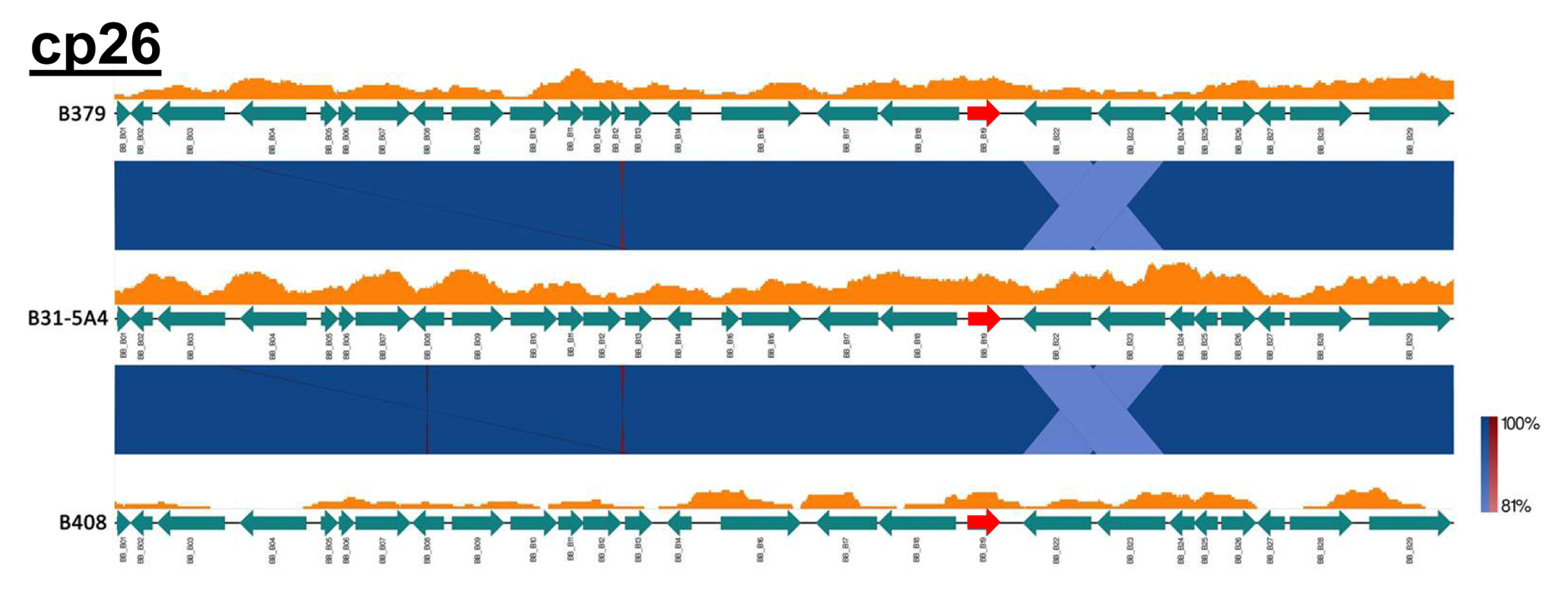

Supplement: FIG S4 [file msystems.00488-22-s0010.jpg]
