# Supplementary material for: Phylogenomic Diversity Elucidates Mechanistic Insights into Lyme Borreliae-Host Association
Source: mSystems. 2022 Aug 8;7(4):e00488-22. doi: 10.1128/msystems.00488-22 (PMC9426539; doi:10.1128/msystems.00488-22)
Supplement: TABLE S1 [file msystems.00488-22-s0001.docx]

**Table S1**

|  | **B31-5A4** | **B379** | **B408** |
| --- | --- | --- | --- |
| **Total base pairs sequenced** | 722,553,355 | 357,749,072 | 568,303,383 |
| **Number of reads** | 72,321 | 49,524 | 78,429 |
| **N50 read length (bp)** | 13,854 | 10,132 | 10,078 |
| **Mean read length (bp)** | 9,990 | 7,223 | 7,246 |
| **Total length of contigs (bp)** | 1513692 | 1417697 | 1392053 |
| **Total length of plasmids (bp)** | 604037 | 496924 | 488804 |
| **Number of total contigs** | 20 | 18 | 18 |
| **Number of chromosomal contigs** | 1 | 1 | 1 |
| **Number of linear plasmids** | 11 | 8 | 10 |
| **Number of circular plasmids** | 9 | 9 | 7 |
| **Number of annotations** | 1527 | 1435 | 1408 |
| **Methylations (per 1000 bp)** | 6.11 | 4.63 | 2.78 |
