# Supplementary material for: Phylogenomic Diversity Elucidates Mechanistic Insights into Lyme Borreliae-Host Association
Source: mSystems. 2022 Aug 8;7(4):e00488-22. doi: 10.1128/msystems.00488-22 (PMC9426539; doi:10.1128/msystems.00488-22)
Supplement: TABLE S2 [file msystems.00488-22-s0002.docx]

**Table S2**

| **Strain** | **Motif Sequence** | **Center Position** | **Modification Type** | **Proportion of Motifs Methylated** | **Number of Motifs Methylated** | **Number of Motifs in Genome** |
| --- | --- | --- | --- | --- | --- | --- |
| **B31-5A4** | CGRKA | 5 | m6A | 0.948 | 2088 | 2202 |
| **B379** | GNAAGC | 4 | m6A | 0.982 | 1871 | 1906 |
| **B408** | GAAGG | 3 | m6A | 0.967 | 1434 | 1483 |
