# Supplementary material for: Phylogenomic Diversity Elucidates Mechanistic Insights into Lyme Borreliae-Host Association
Source: mSystems. 2022 Aug 8;7(4):e00488-22. doi: 10.1128/msystems.00488-22 (PMC9426539; doi:10.1128/msystems.00488-22)
Supplement: TABLE S4 [file msystems.00488-22-s0004.docx]

**Table S4**

| **Strain or plasmid** | **Genotype or characteristic** | **Source** |
| --- | --- | --- |
| *E. coli* | | |
| DH5α | F- Φ80lacZΔM15 Δ(lacZYA-argF) U169 recA1 endA1 hsdR17(rk-, mk+) phoA supE44 thi-1 gyrA96 relA1 λ- | ThermoFisher |
| Rosetta-gami(DE3) | F– ompT hsdSB (rB– mB–) gal dcm lacY1 ahpC (DE3) gor522::Tn10 trxB pRARE (CamR, KanR, TetR) | MilliporeSigma |
| Rosetta-gami(DE3)/pET28a-qC8γ-hPDI | Rosetta-gami(DE3) producing histidine-tagged residues 50 to 228 of the γ chain of quail C8 and residues 18 to 508 of human protein disulfide isomerase. | This study |
| Rosetta-gami(DE3)/pET28a-OmCI-hPDI | Rosetta-gami(DE3) producing histidine-tagged residues 19 to 168 of OmCI and residues 18 to 508 of human protein disulfide isomerase. | This study |
|  |  |  |
| Plasmids | | |
| pET28a | KanR^a^; Histidine-tagged protein expression vector | EMD Milliopore |
| pET28a-qC8γ-hPDI | KanR; pET28a encoding histidine protein residues 50 to 228 of the γ chain of quail C8 with residues 18 to 508 of human protein disulfide isomerase. | This study |
| pET28a-OmCI-hPDI | KanR; pET28a encoding histidine protein residues 19 to 168 of OmCI with residues 18 to 508 of human protein disulfide isomerase. | (1) |

^a^ Kanamycin resistant

**Reference**

1. Kuhn N, Schmidt CQ, Schlapschy M, Skerra A. 2016. PASylated Coversin, a C5-Specific Complement Inhibitor with Extended Pharmacokinetics, Shows Enhanced Anti-Hemolytic Activity in Vitro. Bioconjug Chem 27:2359-2371.
