# Supplementary material for: Phylogenomic Diversity Elucidates Mechanistic Insights into Lyme Borreliae-Host Association
Source: mSystems. 2022 Aug 8;7(4):e00488-22. doi: 10.1128/msystems.00488-22 (PMC9426539; doi:10.1128/msystems.00488-22)
Supplement: TABLE S3 [file msystems.00488-22-s0003.docx]

**Table S3**

| **Nucleotide identity (%)** | **B408 vs. B31-5A4** | | | |
| --- | --- | --- | --- | --- |
|  | **B408** | | **B31-5A4** | |
| 80.25 | BB_G29 | Hypothetical protein | BB_G29 | Hypothetical protein |
| 82.21 | BB_M38 | ErpK protein | BB_M38 | ErpK protein |
| 82.24 | BB_I01 | Hypothetical protein | BB_0408 | Hypothetical protein |
| 82.36 | BB_Q35 | Mlp family lipoprotein | BB_Q35 | Mlp family lipoprotein |
| 82.41 | BB_H09 | Class I SAM-dependent DNA methyltransferase | BB_H09 | Class I SAM-dependent DNA methyltransferase |
| 82.84 | BB_M29 | ERF family protein | BB_O29 | DUF188 domain-containing protein |
| 82.87 | BB_B23 | NCS2 family permease | BB_B22 | NCS2 family permease |
| 83.2 | BB_E02 | Class I SAM-dependent DNA methyltransferase | BB_H09 | Class I SAM-dependent DNA methyltransferase |
| 83.42 | BB_C11 | Site-specific integrase | BB_Q45 | Site-specific integrase |
| 83.5 | BB_E02 | Class I SAM-dependent DNA methyltransferase | BB_E02 | Class I SAM-dependent DNA methyltransferase |
| 83.75 | BB_Q35 | Mlp family lipoprotein | BB_Q35 | Mlp family lipoprotein |
| 84.05 | BB_H09 | Class I SAM-dependent DNA methyltransferase | BB_E02 | Class I SAM-dependent DNA methyltransferase |
| 84.48 | BB_E02 | Class I SAM-dependent DNA methyltransferase | BB_H09 | Class I SAM-dependent DNA methyltransferase |
| 84.97 | BB_H09 | Class I SAM-dependent DNA methyltransferase | BB_E02 | Class I SAM-dependent DNA methyltransferase |
|  | | | | |
| **Percent identity** | **B379 vs. B31-5A4** | | | |
|  | **B379** | | **B31-5A4** | |
| 80.2 | BB_K13 | SIMPL domain-containing protein | BB_K13 | SIMPL domain-containing protein |
| 80.25 | BB_G29 | Hypothetical protein | BB_G29 | Hypothetical protein |
| 81.73 | BB_C03 | Chromosome replication/partitioning protein | BB_L34 | Chromosome replication/partitioning protein |
| 82.24 | BB_I01 | Hypothetical protein | BB_0454 | Hypothetical protein |
| 82.43 | BB_B22 | NCS2 family permease | BB_B23 | NCS2 family permease |
| 82.53 | BB_B23 | NCS2 family permease | BB_B22 | NCS2 family permease |
| 82.63 | BB_Q35 | Mlp family lipoprotein | BB_Q35 | Mlp family lipoprotein |
| 83.41 | BB_Q35 | Mlp family lipoprotein | BB_Q35 | Mlp family lipoprotein |
| 83.7 | BB_L27 | Hypothetical protein | BB_Q34 | Hypothetical protein |
| 84.1 | BB_J34 | Lipoprotein | BB_J34 | Lipoprotein |
| 84.27 | BB_E02 | Class I SAM-dependent DNA methyltransferase | BB_H09 | Class I SAM-dependent DNA methyltransferase |
| 84.73 | BB_L27 | Hypothetical protein | BB_Q34 | Hypothetical protein |
|  |  |  |  |  |
| **Percent identity** | **B379 vs. B408** | | | |
|  | **B379** | | **B408** | |
| 79.71 | BB_K13 | SIMPL domain-containing protein | BB_K13 | SIMPL domain-containing protein |
| 80.38 | BB_I16 | Virulence associated lipoprotein | BB_0397 | Hypothetical Protein |
| 81.62 | BB_E02 | Class I SAM-dependent DNA methyltransferase | BB_H09 | Class I SAM-dependent DNA methyltransferase |
| 81.84 | BB_Q24 | DUF693 family protein | BB_O17 | DUF693 family protein |
| 81.84 | BB_Q24 | DUF693 family protein | BB_O17 | DUF693 family protein |
| 81.84 | BB_Q24 | DUF693 family protein | BB_O17 | DUF693 family protein |
| 82.44 | BB_H09 | Class I SAM-dependent DNA methyltransferase | BB_H09 | Class I SAM-dependent DNA methyltransferase |
| 82.53 | BB_N28 | Mlp family lipoprotein | BB_N28 | Mlp family lipoprotein |
| 82.62 | BB_B23 | NCS2 family permease | BB_B22 | NCS2 family permease |
| 82.65 | BB_E02 | Class I SAM-dependent DNA methyltransferase | BB_E02 | Class I SAM-dependent DNA methyltransferase |
| 82.76 | BB_H09 | Class I SAM-dependent DNA methyltransferase | BB_E02 | Class I SAM-dependent DNA methyltransferase |
| 82.87 | BB_B22 | NCS2 family permease | BB_B23 | NCS2 family permease |
| 82.95 | BB_Q24 | DUF693 family protein | BB_O17 | DUF693 family protein |
| 82.95 | BB_Q24 | DUF693 family protein | BB_O17 | DUF693 family protein |
| 83.1 | BB_Q35 | Mlp family lipoprotein | BB_Q35 | Mlp family lipoprotein |
| 83.13 | BB_A24 | Decorin-binding protein DbpA | BB_A24 | Decorin-binding protein DbpA |
| 83.19 | BB_M34 | Hypothetical protein | BB_M34 | Hypothetical protein |
| 84.05 | BB_E02 | Class I SAM-dependent DNA methyltransferase | BB_H09 | Class I SAM-dependent DNA methyltransferase |
| 84.2 | BB_J34 | Lipoprotein | BB_J34 | Lipoprotein |
| 84.71 | BB_N42 | DUF603 domain-containing protein | BB_S44 | DUF603 domain-containing protein |
