# Supplementary material for: Phylogenomic Diversity Elucidates Mechanistic Insights into Lyme Borreliae-Host Association
Source: mSystems. 2022 Aug 8;7(4):e00488-22. doi: 10.1128/msystems.00488-22 (PMC9426539; doi:10.1128/msystems.00488-22)
Supplement: TABLE S5 [file msystems.00488-22-s0005.docx]

**Table S5**

| **Primer** | **Sequence^a^** | **Amplified DNA fragment** | **Source** |
| --- | --- | --- | --- |
| BBCspZ_prt_fp | GCGGATCCGATGTTAGTAGATTAAATC | *cspZ* without the signal peptide from *Borrelia burgdorferi* | (1) |
| BBCspZ_prt_rp | GCGTCGACCTATAATAAAGTTTGCTTA |  |  |
| BBK32_full_fp | TGGAATCCGACTTAAAATGATTTAA | *bbk32* from *Borrelia burgdorferi* | This study |
| BBK32_full_rp | CATATTTACATATTATGTAGCCTG |  |  |

^a^ Restriction enzyme sites are underlined

**Reference**

1. Marcinkiewicz AL, Dupuis AP, 2nd, Zamba-Campero M, Nowak N, Kraiczy P, Ram S, Kramer LD, Lin YP. 2019. Blood treatment of Lyme borreliae demonstrates the mechanism of CspZ-mediated complement evasion to promote systemic infection in vertebrate hosts. Cell Microbiol 21:e12998.
