# Supplementary material for: Phylogenomic Diversity Elucidates Mechanistic Insights into Lyme Borreliae-Host Association
Source: mSystems. 2022 Aug 8;7(4):e00488-22. doi: 10.1128/msystems.00488-22 (PMC9426539; doi:10.1128/msystems.00488-22)
Supplement: TEXT S1 [file msystems.00488-22-s0006.docx]

**Text S1**

samtools view BC19.subreads.bam \

| awk '{split($23,a,":"); if(and(a[3],64)||and(a[3],128)){print $1}}' \

| tr '/' ' ' | awk '{print $2}' | sort -n | uniq > bad_adapters_holes.txt

bamsieve --blacklist bad_adapters_holes.txt BC19.subreads.bam BC19.filtered.subreads.bam

ccs --log-level INFO -j 16 BC19.filtered.subreads.bam BC19.filtered.ccs.bam

bam2fastq -o BC19.filtered.ccs BC19.filtered.ccs.bam

hifiasm -o BC19.asm -t16 -f0 BC19.filtered.ccs.fastq.g
